# Computationally-accelerated prediction of polyester-melamine coatings degradation to design sustainable organically-coated steels for outdoor applications†

**DOI:** 10.1039/d3ra06744k

**Published:** 2024-06-24

**Authors:** Chris Batchelor, Francisco J. Martin-Martinez, Chris Griffiths, Ian Mabbett, James Smith, Eifion Jewell

**Affiliations:** a SPECIFIC, Swansea University, PMRC 24-26 Mardon Park, Central Ave Port Talbot SA12 7AX UK c.p.batchelor@swansea.ac.uk; b Swansea University Chemistry Department Singleton Park, Sketty Swansea SA2 8PP UK; c Becker Industrial Coatings Ltd Goodlass Road Liverpool L24 9HJ UK

## Abstract

This work implements computational chemistry as a screening tool to aid in the coating and resin formulation process. Conceptual Density Functional theory (DFT) reactivity descriptors like the global chemical hardness and the dual descriptor Fukui function identify the tendency of polyester-melamine coatings to undergo electrophilic and nucleophilic attack during weathering exposure. Coatings were subjected to natural and accelerated weathering tests, with periodic infrared spectroscopy, colour, and gloss measurements to assess for the degree of changes brought about through photodegradation. It was found that the number of attack sites in the atomistic models, when weighted as a function of the polyester : crosslinker ratio, effectively ranked the degradation of different coating systems upon weathering. This ranking matched the performance of the coatings subjected to both accelerated and natural weathering, showing affinity with naturally weathered samples, and matching in all areas. The results were shown to demonstrate significant correlation, being over *R*^2^ = 0.8 for 7 of the 8 measured areas, and greater than *R*^2^ = 0.9 for 6 compared areas. Comparison of computationally derived and experimentally acquired results showed that the performance of naturally weathered samples was matched across all areas by the computational rankings, showing superior correlation than that observed between natural and accelerated weathering tests. This indicates that the method utilised within this work provides a novel, cost-effective alternative to evaluate the projected performance of selected coatings, while enabling a computationally accelerated platform for more sustainable low-degradation coatings without the requirement of long-term weathering tests.

## Abstract 2

The study combines computational chemical modelling and practical weathering investigations, finding that computational analysis matches real-world weathering degradation across all investigated areas in a much shorter time than weathering tests.

## Graphical abstract

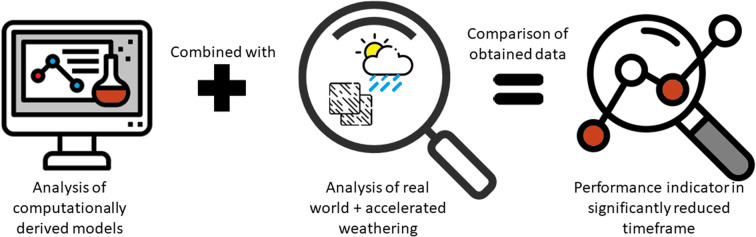

## 1 Introduction

Environmental concerns and sustainability awareness in the built environment, are calling for more durable, resilient, and sustainable coatings. Organically coated steels are used in a wide range of outdoor applications including building cladding and roofing. During service, the coatings are exposed to harsh environmental conditions such as variations in humidity and temperature along with ultraviolet (UV) radiation exposure. These conditions contribute to a reduction in the aesthetic value of the coating, as well as to a decrease in the chemical resistance, which limits the service life of the coating. Thus, fast testing of the degradation induced by humidity combined with UV radiation is essential in the development of more durable and sustainable organically coated steel.

To this end, accelerated weathering tests are typically used to measure the combined effects of UV radiation and humidity on the degradation of organic coatings. These tests are run to standards that make it possible to draw comparisons of coating performance depending on the formulation or substrate choice. It is commonly accepted that 2000 hours of accelerated weathering is approximately equivalent to 2 years of Natural weathering, providing comparable results in months rather than years.^1^ However, they have been shown to offer poor comparisons to natural weathering conditions,^1,2^ which limits their application. Another issue with accelerated weathering is that they must run for thousands of hours before results are available, increasing the cost, and slowing down the research process. While UV-A, B, and C bulbs are all available for use within accelerated weathering cabinets and focus on different ranges of the UV region, UV-A is the most commonly experienced for external coatings on the surface of the Earth.^1^

The study of polymer degradation induced by UV radiation is well-documented within the literature.^3–5^ Absorbing UV radiation brings about photo-oxidative degradation which in turn causes the breakdown of the hydrocarbon backbone of the polymer and produces free-radicals. As free-radicals are self-propagating, if the environmental conditions remain the same, they induce a rapid degradation of the coating system once the initial stage of free-radical formation is reached. Furthermore, free-radicals promote further oxidation reactions that in turn produce additional free-radicals, subsequently damaging several coating layers. This cascading reaction leads to chain scission and a significant reduction in the molecular weight of the polymer, which compromises the performance of the coating under working conditions.^6^ Moreover, in addition to radically initiated photodegradation there are hydrophilic areas, such as ester linkages, crosslinker rich zones and pigment rich areas, which are susceptible to hydrolysis. Removing these zones *via* interactions with water, in the case of acid catalysed hydrolysis, causes an increase in surface roughness, which is observed by a decreased gloss value. These craters in the coating surface will then allow for deeper subsequent moisture penetration, bringing further potential damage to the coating system. Also, hydrolysis causes the polymer bonds to directly break down the coating, or it targets the polymer–substrate interface to cause delamination of the coating. It is also known that hydrolysis is particularly damaging to the links between the polymer and melamine crosslinkers,^7,8^ and chemical functional groups of special interest. Therefore, moisture-enhanced photodegradation is known to be exceptionally harmful to coating systems, as it has a synergistic effect of free-radical formation, increase in surface roughness, delamination and hydrolysis.^9–11^

As a case study, this paper focuses on polyester-melamine coatings, a popular choice for coil coating steels with a wide range of outdoors and indoors uses. The monomers used to develop the coatings were Hexahydro Phthalic Anhydride (HHPA) and Cyclohexane Dicarboxylic Acid (CHDA). Both are cycloaliphatic structures used to generate the resins that make up the bulk of the final coatings, therefore being the main responsible of the properties of the final coating.^12^ While coating systems typically include radical scavengers such as hindered amine light stabilisers (HALs) and UV absorbers for their sacrificial protective properties that have been shown to extend the life of coatings,^13^ they have been omitted from this investigation so that the inherent weatherability of the coatings could be determined without the influence of additives. Additionally, HHPA has come under scrutiny as a potential hazard in some industries in its powder form, making it prudent to investigate safer alternatives.^14^ This fact further highlights the need of a computationally accelerated framework for coatings degradation that facilitates the discovery of more sustainable monomers.

To accelerate the design and selection of new materials for organic coatings, computational chemistry methods, atomistic simulations, and high-throughput supercomputing can assist in the prediction of the relative performance of coatings when in service, easing the cost of experimental testing and accelerating material discovery. While computational chemistry and atomistic modelling has been in use since before the 1950's, it was with the increasing popularity of computers and developments in computational resources that it became widely used. Nowadays, it is possible to perform simple calculations using the average personal computer, let alone more specialised supercomputing centres. As the models increase in complexity there is a corresponding increase in the computational cost, which translates into computation time and cost for any calculation to be completed. To circumvent this, supercomputing facilities like supercomputing Wales (SCW) implement high-throughput computing (HTC), and high-performance computing (HPC) to enable lower-cost fast calculations concurrently.^15^

In fact, density functional theory (DFT) allows for reactivity calculations that provide a comparative analysis of different coating candidates, easing the development process with results available within days rather than months or years. It also offers insightful information to coating formulators, potentially maximising the number of suitable coatings produced, while reducing excess in chemical consumption and production times. DFT is rooted in quantum mechanics and it provides information on the ground state and chemical properties of molecules and materials,^16–19^ including thermodynamic stability, reactivity, and light absorption. It is therefore a suitable theoretical framework to study degradation and corrosion in organic coatings.

The main aims of this paper are to prove the suitability of conceptual DFT reactivity descriptors to accurately predict the performance of organic coatings in accelerated and natural weathering tests. As a case study, we investigate the effect of CHDA and HHPA monomers, with different crosslinker ratios on the degradation resistance of coatings, while simultaneously validate DFT predictions. A novel method is used during the interpretation of the computational results, whereby the computational data is weighted as a function of the resin : HMMM ratio, this makes it possible to investigate coatings with the same formula in different ratios while minimising the potential introduction of errors by maintaining a simplistic approach. This would potentially circumvent the need for long-term weathering tests and have a significant impact on testing procedures and coating innovation going forward, as well as it would set the floor for high-throughput calculations of monomers and additives to generate large data sets. The need for more sustainable coating materials in the built environment, and the interest in decarbonizing the steel industry, calls for computationally accelerated strategies that improve the current degradation testing at low cost. Furthermore, the rise of artificial intelligence (AI) and machine learning (ML) models is further revolutionizing materials discovery, but the accuracy of AI predictions deeply relies in the quality of the training data. Thus, experimentally validated data like the one produced by the computational framework suggested in the current work, will provide reliable data sets for ML training in the future.

## 2 Methodology

### 2.1 Materials and manufacturing

The coatings under study were developed using proprietary formulations by Becker Industrial Coatings Long Term Development Group. Pigmented coating systems were applied to pre-treated steels. Free-films were generated through the application of clear-coatings to a polytetrafluoroethylene (PTFE) treated sheet and then cured in an oven. All coatings were cured in a Bird industrial curing oven for 35 seconds to reach a Peak Metal Temperature (PMT) of 232 °C.

To investigate the inherent weathering resistance of cycloaliphatic monomers, the resin formulations were made so that either HHPA or CHDA was the main polyester (PE) monomer base, with neopentyl glycol (NPG), trimethylol propane (TMP), ethyl glycol (EG), and hexanediol (HD) as additional resin components. Hexa methyl methoxy melamine (HMMM) was used as a crosslinker during coating development, while pigments were also added to generate a brown coating to emulate a panel for exterior use, which are commonly coloured. All coatings were applied to pre-primed steel using a manual wire bar coater to produce coatings with a 25 μm thickness before being cured to reach a peak metal temperature (PMT) of 232 °C for 35 seconds, simulating curing conditions on a coil line. A summary of the coating systems under study can be found in Table 1. The coating codes used through this paper indicates the main constituent of the coating and the resin : HMMM ratio, *e.g.*, CHDA*8020Br describes a CHDA-based coating with a ratio resin : HMMM of 80% to 20% (following the standard industrial method), while Br denotes the colour of the sample, in this case, chocolate brown. The resin : HMMM ratio does not include the addition of pigments or other additives but is a direct representation of the amount of resin added to the formulation compared to the crosslinker.

**Table 1:** Summary of coatings used in weathering tests, including molecular weight, *T*_g_, resin : crosslinker coating ratio (up to 100%), hydroxyl value, and main components within the formulation

Coating code	Resin : crosslinker ratio	*T*_g_ (°C)	Straight chain (%)	Hydroxyl value (mg_KOH_ g^−1^)	Mn (number average molecular weight) g mol^−1^	Main resin components
HH*8020Br	80 : 20	32	17.8	53	4082	HHPA, NPG, TMP, EG, HD
HH*8515Br	85 : 15	29	17.8	53	4082	HHPA, NPG, TMP, EG, HD
CHDA*8020Br	80 : 20	30	16.3	54	4212	CHDA, NPG, TMP, EG, HD
CHDA*8515Br	85 : 15	29	16.3	54	4212	CHDA, NPG, TMP, EG, HD

The resin formulations were made to attain an average molecular weight (Mn) of 4000–4300 g mol^−1^ and a hydroxyl excess value of 52–54 mg_KOH_ g^−1^.^12,20^ To minimise the influence of the resin formulations over weathering performance results, the straight chain percentage was kept as uniform as possible across the different coatings by controlling the additions of EG and HD. The straight chain percentage refers to the percentage weight addition of straight chain components within the resin system.

The number average molecular weight (Mn) was measured *via* an Agilent Gel permeation chromatograph during the final stages of resin development. This analysis made it possible to ensure molecular weights were kept as similar as possible. The midpoint glass transition temperature (*T*_g_) for the different coating systems were determined through Differential Scanning Calorimetry (DSC) with a Mettler Toledo DSC1 and a temperature ramp rate of 10 °C min^−1^.

### 2.2 Experimental details

Samples were exposed to natural weathering and accelerated tests as described in BS EN ISO 11507:2007. Naturally exposed samples were placed Southward facing at a 45° angle at a weathering site in Florida for 2 years. Gloss and colour measurements taken every 6 months, with Fourier Transformed Infrared (FTIR) scans obtained every 12 months (natural weathering) using a PerkinElmer Spectrum 100 with a Specac Attenuated Total Reflectance (ATR) attachment. Samples subjected to accelerated weathering were exposed with UV-A radiation at 60 °C for 4 hours followed by condensation at 40 °C for 4 hours through the use of Q-lab accelerated weathering cabinet fitted with UV-A 340 nm bulbs that are designed to simulate direct sunlight. After every 250 hours gloss and colour measurements would be taken, with FTIR measurements obtained following every 500 hours of exposure, measurements were taken in the same location on each sample using a Specac ATR attached to a PerkinElmer FTIR 100 spectrometer.

FTIR bands were normalised against the baseline area value for the CH_2_–CH_3_ band of each sample. The CH band was chosen as it is representative of the hydrocarbon backbone and it is expected to remain consistent until the final stages of coating degradation. The primary focus of FTIR analysis was analysis of hydroxyl (3800 cm^−1^ to 2100 cm^−1^) and carbonyl (1800 cm^−1^ to 1650 cm^−1^) activity, as degradation products are known to present themselves within them. This would mean that an activity increase within these regions would correspond to coating degradation.^21–23^ It is known that the hydroxyl region can be influenced by other factors such as post-curing reactions during weathering and would influence the normalised average change in hydroxyl activity, this is not observed in the carbonyl peak due to being a narrow peak.

Gloss measurements were obtained using a handheld GM1 Glossmeter that utilised a 60° contact angle with an error value of ±1.2 gloss units. For statistical significance, 5 gloss measurements were taken for each sample, with the average value being calculated and converted into a percentage value relative to the baseline gloss measurements, thus highlighting gloss loss during weathering. As gloss is an indicator of surface roughness, a decreasing gloss value directly corresponds to increasing surface roughness.^24^ Colour analysis was performed with a Colour X-rite Spectrolino in specular included mode to perform CIEL*a*b* measurements, which provide measurements for light to dark (L*), range from magenta to green (a*), and blue to yellow (b*). These measurements were in turn used to calculate Δ*E* (the “distance” between the initial and post exposure colours) for the coatings relative to their colours prior to exposure, the calculation for which is shown in eqn (1).^25^1
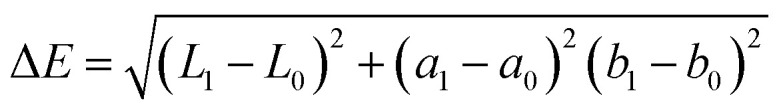
where (*L*_1_ − *L*_0_) identifies the change in lightness between post- and pre-exposure, (*a*_1_ − *a*_0_) is the shift on the red-green spectrum between post-and pre-exposure, and (*b*_1_ − *b*_0_) measures the change on the yellow to blue spectrum between post- and pre-exposure.

Non-pigmented free-standing films were developed to investigate the inherent UV absorbance of HHPA and CHDA polyester-melamine coatings. All UV measurements were performed using a PerkinElmer UV-Vis-NIR Lambda 950 with a 150 nm integrating sphere, which measured from 200 nm to 2500 nm at increments of 10 nm in transmission mode.

### 2.3 Computational details

DFT^19^ calculations were performed using ORCA computational chemistry package at the DFT standard calculation parameters of 298 K and 1 atm of pressure.^26,27^ Geometry optimizations of all the different model molecules under study were performed using the def2-TZVPP triple zeta basis set and the commonly used B3LYP hybrid functional.^28,29^ The B3LYP hybrid functional was chosen due to its record of accurate predictions of molecular structures and other properties, which has led to it being one of the most widely used DFT functional. The def2-TZVPP triple zeta basis set was chosen as it is a recommended basis set for use with DFT calculations, as well as resulting in the convergence of geometries and energies at the DFT level.^30,31^ The initial geometries for the DFT calculations were built with Avogadro software, and the optimised structures were visualised using Visual Molecular Dynamics (VMD).^32,33^

**Fig. 1:**
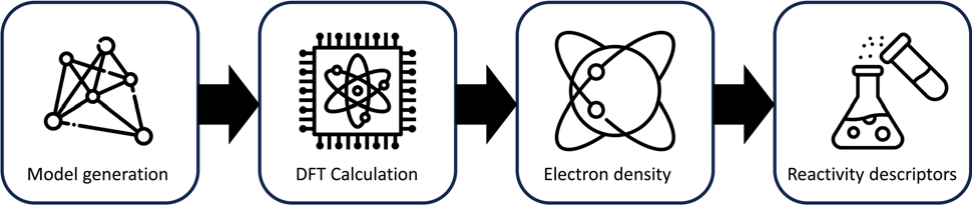
Breakdown of the computational chemical modelling process.

All calculations were performed at Supercomputing Wales (SCW) facilities.^15,26^ A breakdown of the process by which the computational models were generated is laid out in Fig. 1.

The global chemical hardness reactivity descriptor (*η*) was calculated using eqn (2).

2 *η* = ½(*I* − *A*)

where *I* is the ionization energy and *A* is the electron affinity. To estimate the values of *I* and *A*, single point calculations were performed on the optimised structures of each molecule after removing and adding one electron to the neutral molecules, the corresponding energies were then estimated as shown in eqn (3) and (4).^17,28^

3 *I* = *E*_N−1_ − *E*_N_

4 *A* = *E*_N+1_ − *E*_N_

where *E*_N−1_ is the energy of negatively charged molecules, *E*_N+1_ the energy of positively charged molecules, and *E*_N_ = energy of neutral molecules. To evaluate an approximated but faster way to calculate the global chemical harness, *I* and *A* were estimated from the energies of the highest occupied molecular orbital (HOMO) and lowest unoccupied molecular orbital (LUMO), respectively, following Koopman's theorem. Within this approximation, *I* is calculated as the negative energy of the HOMO (−*ε*_HOMO_), while *A* is calculated from the energy of the LUMO (−*ε*_LUMO_), with *ε*_HOMO_ and *ε*_LUMO_ being the energies of the HOMO and LUMO orbitals respectively, with all calculated energies being vertical for the ground state optimised geometries of the investigated molecules.^17,28^

The local reactivity is evaluated by the dual descriptor of the Fukui function. The Fukui function describes the electron density of a molecule after the removal (eqn (5)) or addition (eqn (6)) of electrons. The analysis of the electron density following these changes in electrons number make it possible to identify qualitatively the areas of a molecule that would undergo electrophilic or nucleophilic attacks upon chemical reaction. In fact, the dual descriptor of the Fukui function (eqn (7)) describes both the positive and negative Fukui functions and it makes it possible to characterise the local chemical reactivity of a molecule.^19,34^

5 *f*^−^(*r*) = *ρ*_*N*_(*r*) − *ρ*_*N*−1_(*r*)

6 *f*^+^(*r*) = *ρ*_*N*+1_(*r*) − *ρ*_*N*_(*r*)

7 *f*^(2)^(*r*) = *f*^+^(*r*) − *f*^−^(*r*)

where *N* is the total number of electrons, and *ρ*_*N*_(*r*), *ρ*_*N*−1_(*r*), *ρ*_*N*+1_(*r*) are the electron densities of the neutral, positive and negative molecules respectively. These areas susceptible to electrophilic or nucleophilic attack were highlighted using Colour ID to differentiate the sites and isosurface values of 0.01/−0.01, the colours and isosurface values were kept constant across all investigated models. These values were determined through a trial-and-error approach as the greatest values without causing significant overlap between attack sites. All of the models were inspected from multiple perspectives to ensure that the attack sites were correctly counted.

To make it possible to investigate resin : HMMM systems with the same components in different ratios, it was decided that the results would be weighted as a function of the resin : HMMM ratio. This means that when concerning an 80 : 20 coating system the number of reactive sites is counted and multiplied by 0.8 (representative of the 80 percent polyester ratio), while all the relative crosslinker containing components have the number of reactive sites added up and multiplied by 0.2, with a similar process being followed for the 85 : 15 coatings. To our knowledge, this is the first attempt to utilise conceptual DFT reactivity descriptors to predict relative steel coating performance in real-world weathering tests.

## 3 Results and discussion

### 3.1 Experimental testing and characterisation

An initial study to investigate the influence of HHPA and CHDA on the weathering durability of coating systems was performed by subjecting several HHPA- and CHDA-based coatings with varying crosslinker ratios to accelerated and natural weathering tests. The tests focused on investigating the coatings degradation from the changes in normalised hydroxyl and carbonyl activity as identified during FTIR analysis, as well as from the changes in colour and gloss value. Subjecting the coatings to accelerated and natural weathering tests also enabled a comparison of coatings performance under these two weathering methods.

**Fig. 2:**
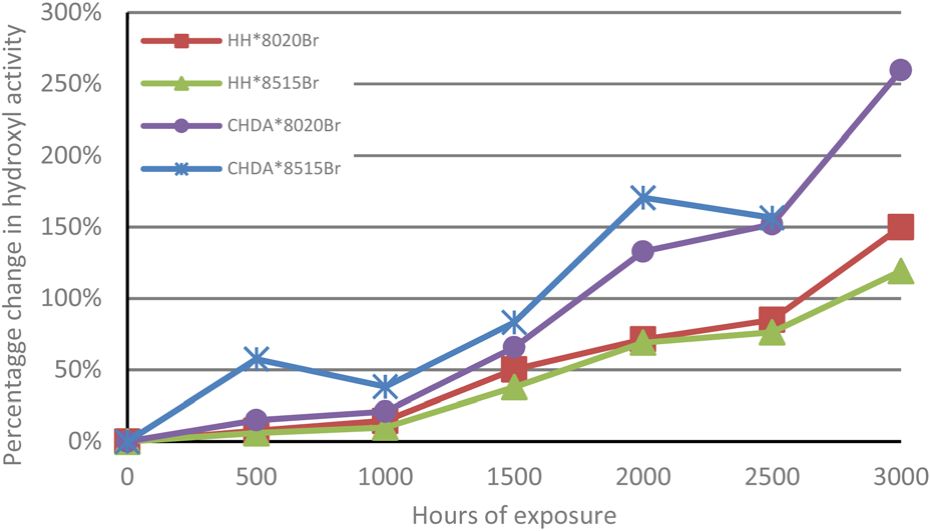
Comparison of average percentage change in normalised hydroxyl activity of samples subjected to accelerated UV-A weathering (100% at *t* = 0).

The change in normalised hydroxyl activity for the samples that were subjected to accelerated UV-A weathering are illustrated in Fig. 2. The degradation is identified by the percentage change in hydroxyl activity reaching a peak after 2000 hours exposure, followed by a decrease at 2500 hours, which represents high levels of degradation and coating removal. The poorest performing coating was CHDA*8515Br, as evidenced by its removal from testing after 2500 hours due to significant degradation resulting in removal of the coating system. CHDA*8020Br was the second poorest performing, followed by HH*8020Br, and HH*8515Br. Overall, CHDA coatings demonstrate significantly greater increases in hydroxyl activity during weathering compared to coatings based on HHPA, which can be representative of increased rates of degradation and greater moisture absorption.

**Fig. 3:**
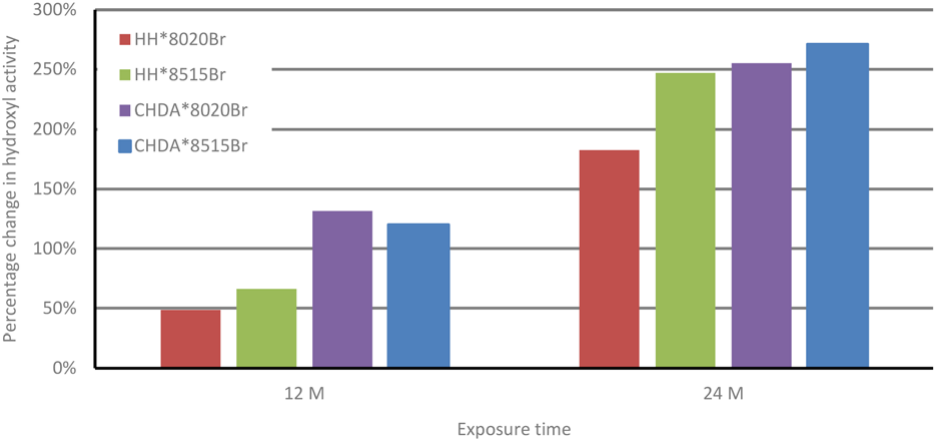
Comparison of average percentage change in hydroxyl activity of HHPA- and CHDA-based coatings in natural weathering (100% at *t* = 0).

Fig. 3 shows the percentage change in normalised hydroxyl activity of the coatings following 12 and 24 months of natural weathering. While HHPA-based coatings again outperform those based on CHDA, the 80 : 20 coatings show superior weathering resistance compared to 85 : 15 coatings. This could be attributed to several factors impacting coatings subjected to natural weathering, which are not replicated in the accelerated weathering tests, *i.e.*, temperature fluctuations, acid rain, or biological fouling.^1,2,35,36^ It is possible that *T*_g_ also plays a factor in the observed differences between accelerated and natural weathering, as coatings subjected to natural exposure are likely to spend less time above their *T*_g_ than those coatings subjected to accelerated weathering.

**Fig. 4:**
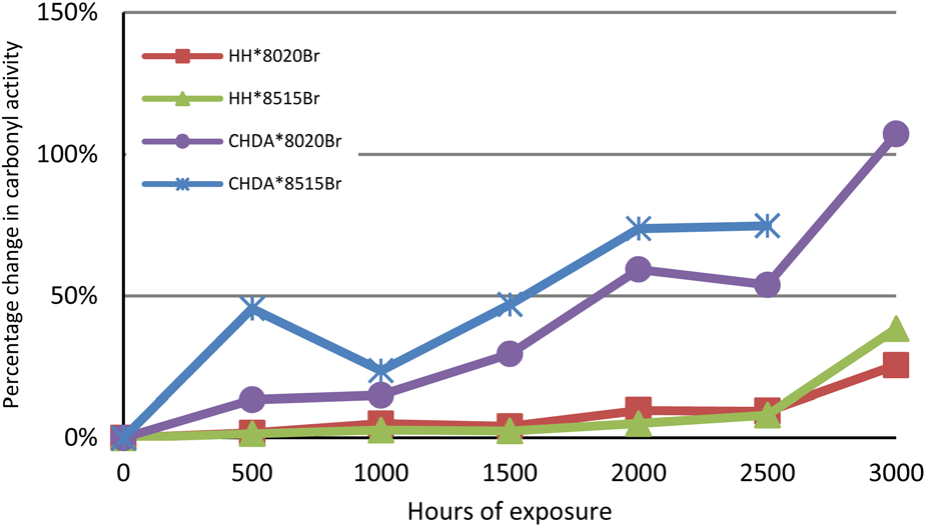
Comparison of average percentage change in normalised carbonyl activity of samples subjected to accelerated UV-A weathering.

**Fig. 5:**
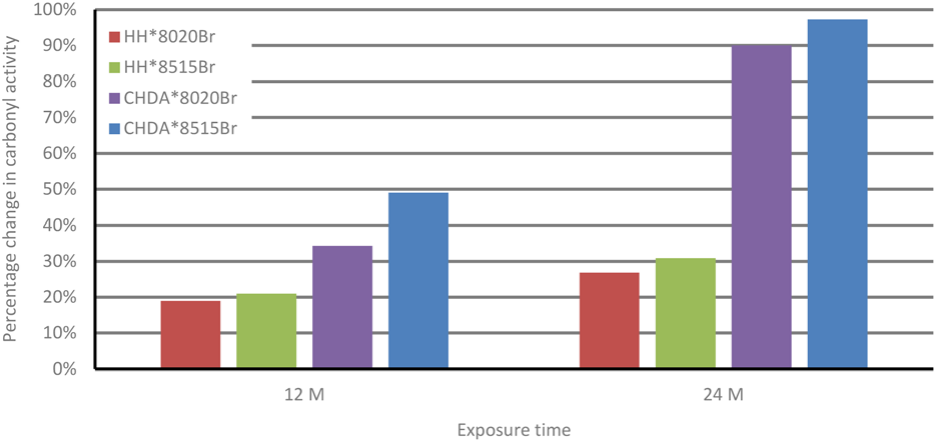
Comparison of average percentage change in carbonyl activity of HHPA and CHDA coatings in natural weathering.

The changes in percentage change in normalised carbonyl-based coatings exhibit significantly greater changes in normalised carbonyl activity compared to their HHPA-based counterparts, with CHDA*8515Br experiencing the greatest increases in activity. Activity for coatings subjected to accelerated and natural weathering are shown in Fig. 4 and 5, respectively. An analysis of the data in Fig. 4 shows that HH*8020Br exhibits the smallest increase in carbonyl activity, while CHDA*8515Br undergoes the greatest increase.

Fig. 5 and 7 follow a similar pattern in terms of performance, with HH*8020Br showing the smallest increase in activity, followed by HH*8515Br, CHDA*8020Br, and CHDA*8515Br. The data in Fig. 2–4 show that HHPA- and CHDA-based coatings under natural weathering (Fig. 4) also follows a similar pattern to that observed in Fig. 5 and 7.

**Fig. 6:**
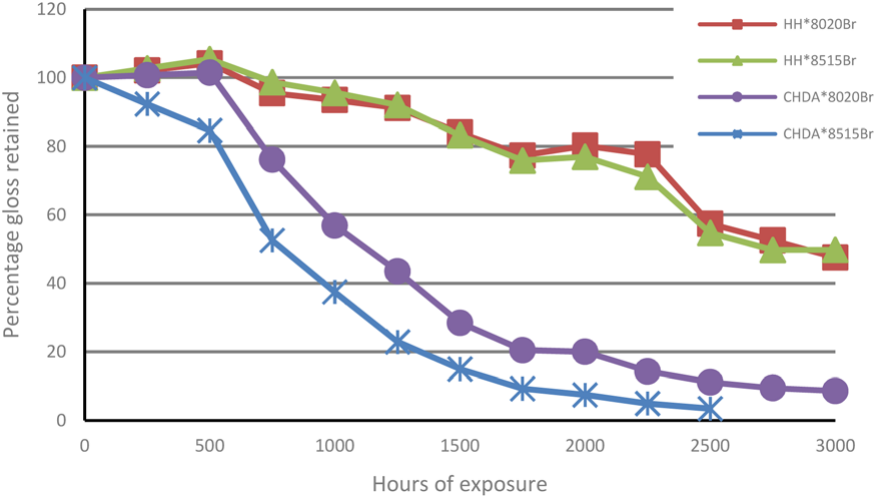
Comparison of average percentage gloss retention (accelerated weathering).

**Fig. 7:**
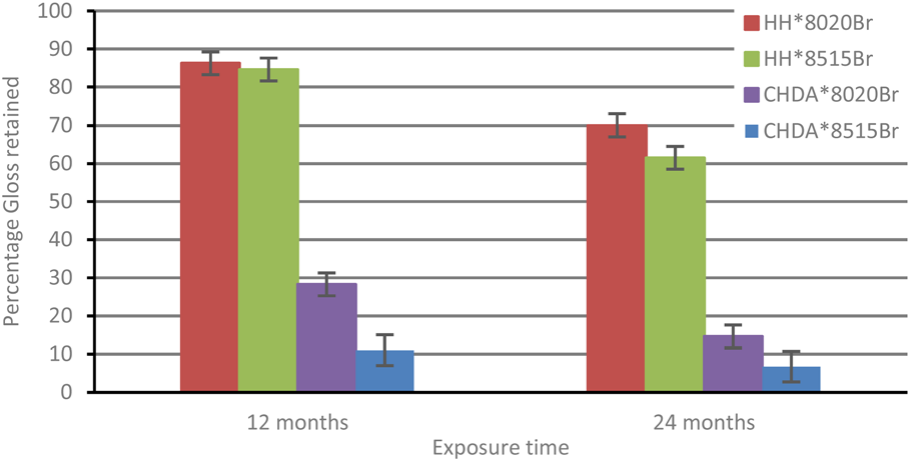
Comparison of average percentage gloss retention (natural weathering).

The percentage of gloss retention of samples subjected to accelerated and natural weathering are illustrated in Fig. 6 and 7, respectively. Fig. 6 shows that all coatings experienced a decrease in gloss, with the CHDA-based coatings exhibiting significantly greater decreases in gloss during accelerated UV-A accelerated weathering, compared to HHPA coatings. CHDA*8515Br shows the most significant decrease in gloss prior to its removal after 2500 hours, while the HHPA coatings showed very similar final gloss values after 3000 hours of weathering.

**Fig. 8:**
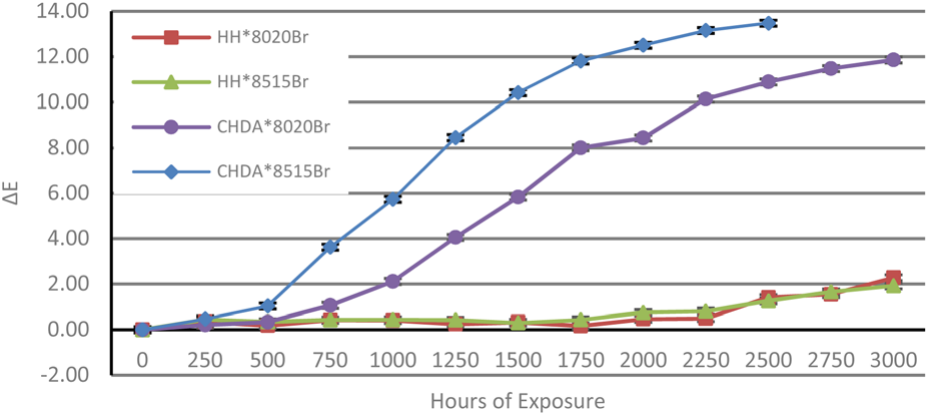
Δ*E* over time for samples subjected to accelerated UV-A weathering.

**Fig. 9:**
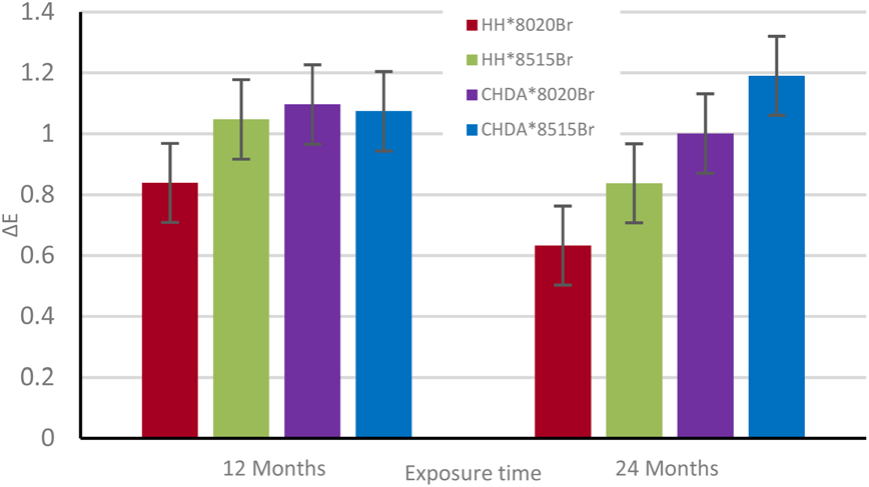
Δ*E* over time for samples subjected to natural weathering.

Δ*E* of the samples was calculated from data obtained with CIEL*a*b* measurements to illustrate the total colour change after accelerated and natural weathering cycles. The results are shown in Fig. 8 and 9, respectively. Fig. 8 shows that CHDA-based coatings undergo significantly greater change in Δ*E* than HHPA-based coatings, with CHDA*8515Br showing the greatest degree of colour change prior to its removal at 2500 hours. HH*8020Br and HH*8515Br show very similar Δ*E* values up to 3000 hours.

Fig. 9 shows the Δ*E* of samples exposed through natural weathering. While there is an overall positive Δ*E* following 2 years of exposure, there is also a decrease after 12 months. As these samples are washed periodically, it is possible that a fluctuation in the observed Δ*E* is a consequence of repeated washings. As with the samples subjected to accelerated weathering, the CHDA-based coatings showed poorer performance compared to those with HHPA, with the 85 : 15 resin : HMMM ratio coatings showing a more significant Δ*E* than the 80 : 20 variants.

Table 2 shows the change in CIEL*a*b* measurements prior to exposure and final analysis. The most significant change in measurements occurs during accelerated weathering. CHDA-based coatings exhibit a significant change within the L*, which corresponds to increased lightness. This fact suggests a potential chalking and bleaching of the overall coating. It is possibly a consequence of the greater number of changes between UV-A radiation and water condensation cycles, which would in turn facilitate a greater number of instances of moisture-enhanced photodegradation, which CHDA-based coatings may be more vulnerable to.

**Table 2:** Differences between initial and final CIEL*a*b* measurements for coating systems under study

	Coating code	L*	a*	b*
Accelerated UV-A	HH*8020Br	1.57	−0.98	−1.33
	HH*8515Br	1.10	−0.89	−1.30
	CHDA*8020Br	9.77	−4.29	−5.18
	CHDA*8515Br	11.73	−4.71	−5.15
Natural weathering	HH*8020Br	0.23	−0.14	−0.58
	HH*8515Br	0.38	−0.27	−0.70
	CHDA*8020Br	0.26	−0.50	−0.82
	CHDA*8515Br	0.36	−0.70	−0.89

As previously mentioned, the absorbance spectra of the two PE resin systems were anticipated to be similar. This was investigated by generating clear coat free-film coatings, which were applied to PTFE sheets and cured before being peeled off. Free-films made it possible to determine transmission through the coating, which was then used to calculate absorbance values. Non-pigmented free-films were chosen to gain a greater understanding of the absorbance values of the coatings themselves, without influence from pigments. The similarity in UV absorbance values is proven by the results in Table 3 which shows the calculated absorbance values across the UV region, the specific UV-A, B, and C regions, as well as ranking the values from lowest to highest absorbance. The data shows that all the free-films exhibited similar absorbance profiles, with the lower HMMM ratio coatings exhibiting lower absorbances compared to the 80 : 20 counterparts. This proves that the significant differences in performance cannot be explained by absorbance of UV radiation. It was therefore hypothesised that the difference in sensitivity to hydrolytic interactions could be the cause of the greater degradation of CHDA-based resin : HMMM coating, which was investigated through computational means.

**Table 3:** Breakdown of the UV absorbances of investigated resin : HMMM films

Coating code	Full UV area	UV-A (400–320)	UV-B (315–280)	UV-C (280–100)	Full UV rank	UV-A rank	UV-B rank	UV-C rank
HH*8020	179.27	7.06	14.43	157.77	4	3	3	4
HH*8515	172.45	6.24	11.65	154.56	1	1	1	1
CHDA*8020	179.24	7.06	15.68	156.49	3	4	4	3
CHDA*8515	173.34	6.58	11.96	154.80	2	2	2	2

### 3.2 Density functional theory calculations

**Fig. 10:**
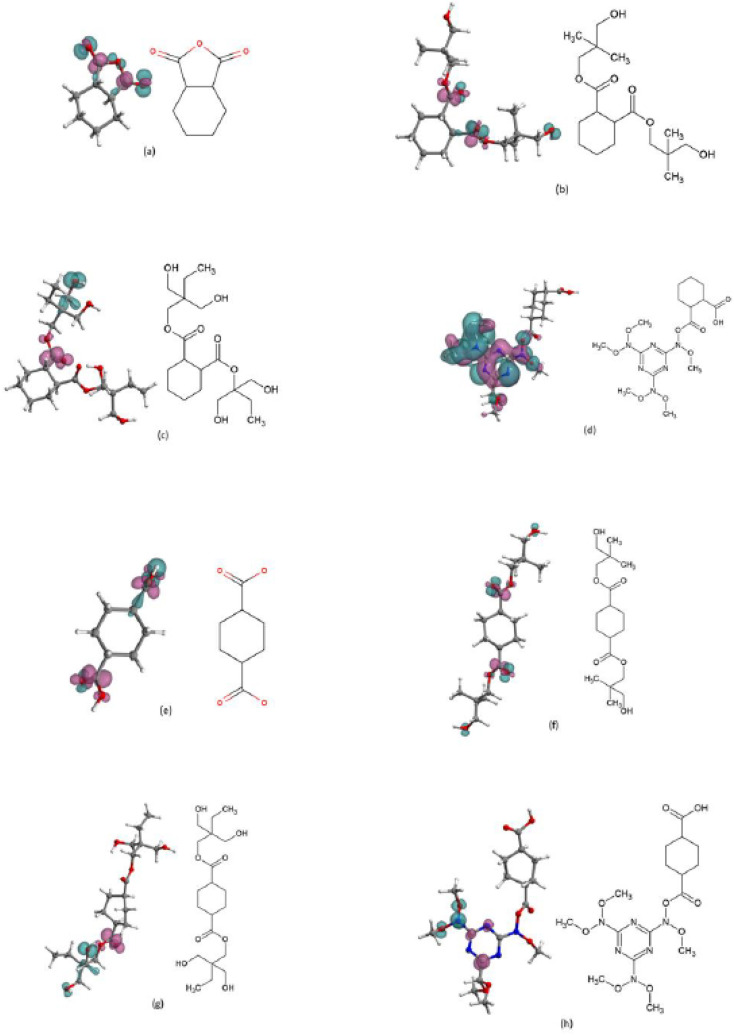
Dual descriptor of the Fukui function alongside the model molecules of the main components of coatings formulations, HHPA (a), HHPA and NPG (b), HHPA and TMP (c), HHPA and HMMM(d), CHDA (e), HHDA and NPG (f), CHDA and TMP (g), and CHDA and HMMM (h). The dual descriptor of the Fukui function is plotted on isosurface of 0.01/−0.01. The areas that undergo electrophilic attack are coloured in green, and the areas that would undergo nucleophilic attack are coloured in purple.

To evaluate the suitability of conceptual DFT reactivity descriptors, *i.e.*, the global chemical hardness, and the dual descriptor of the Fukui function, to correlate weathering tests and therefore predicting steel coatings degradation, we performed DFT calculations on model molecules that represent the HHPA, CHDA, NPG, TMP, and HMMM monomers. As the resins are hydroxyl rich, the possibility of the cycloaliphatic acid and the HMMM joining together is low in practice, but the model was included to investigate inherent vulnerabilities to electrophilic and nucleophilic attack of the structure if this were to occur. Fig. 10 shows the chemical structure and the dual descriptor of the Fukui function for the different model molecules considered, while Table 4 shows the number of reactive sites (electrophilic or nucleophilic) in those molecules together with the values of the global chemical hardness.

**Table 4:** Number of sites susceptible to electrophilic and nucleophilic attack according to the dual descriptor of the Fukui function, and the values of the global chemical hardness

Model	Number of electrophilic sites	Number of nucleophilic sites	Approximate chemical hardness (eV)	Exact chemical hardness (eV)
HHPA	8	8	4.48	4.62
HHPA NPG	8	8	3.84	3.54
HHPA TMP	9	3	3.63	3.67
HHPA HMMM	9	10	3.66	4.08
CHDA	8	12	3.82	3.94
CHDA NPG	12	10	3.86	3.67
CHDA TMP	7	6	3.69	3.09
CHDA HMMM	6	6	3.72	4.08

The choice was made to focus computational work on the same components that comprised the main components of the resins used to develop the coatings investigated in Section 3.1. As the focus was to investigate a simplistic and quick way of screening resin systems it was decided to investigate the individual components of the system and examples where one component was joined to another. Avoiding modelling entire polymer systems made the process faster and significantly reduced the associated computational costs.

CHDA and CHDA-based molecules exhibited the largest total number of reactive sites, followed by HHPA. The results included in Table 4 also shows that HHPA-based and CHDA-based structures have similar numbers of electrophilically reactive sites, with the exception of CHDA-NPG. CHDA-based structures also demonstrate a greater number of reactive sites that are susceptible of nucleophilic attacks. These results imply that CHDA-based polymers are expected to be more reactive under water conditions, which would correlate with the results observed in the weathering tests.

Thus, the results listed in Table 4 supported the hypothesis that the performance of a group of coatings in terms of percentage gloss loss, overall colour change, and changes in hydroxyl and carbonyl activity can be ranked through weighted comparison of the number of electrophilic and nucleophilic attack sites within the models.

Our study suggests that gloss loss could be predicted as a function of the number of nucleophilic reactive sites, as water is a known nucleophile and coating surface reactions with water would remove the coating components within the more hydrophilic regions at the coating surface through “drop out”, promoting surface roughness and causing a corresponding decrease in gloss values.

The observed changes in hydroxyl and carbonyl activity are indicative of the development of degradation product, which are formed through the degradation of the coating system during breakdown of the polymer in the form of free radicals, which in turn would lead to further polymer degradation. Changes in the average percentage change in hydroxyl and carbonyl activity are indicative of degradation product formation, particularly free-radicals that in turn bring about further damage to the polymer chain.

Both the approximate and exact chemical hardness values are laid out in Table 4, the purpose of this was to determine if there was a significant difference between the sets of values. There is a significant difference between the approximate and exact chemical hardness values. The exact values require further calculation, but due to the increased accuracy that this offers it was decided to focus on the exact chemical hardness values.

### 3.3 Conceptual DFT and experimental weathering tests

Table 5 illustrates a comparison between the results of the conceptual DFT reactivity descriptors, 2 years of natural weathering, and 2000 hours of accelerated weathering. It is commonly accepted that 2000 hours of accelerated weathering exposure being commonly used as an equivalent for 2 years of natural weathering.^1^ In fact, accelerated weathering after 2000 hours showed a correlation in gloss and colour retention when compared to samples subjected to natural weathering for 2 years. However, there were some differences in the rankings of coatings for changes in hydroxyl and carbonyl activity, which correspond to the formation of degradation products. Showing correlation between weighted computationally calculated results and data obtained through more traditional weathering methods would facilitate a significant reduction in testing times.

**Table 5:** Summary of experimental performance parameters, including final percentage gloss retention, Δ*E*, percentage change in hydroxyl activity, percentage change in carbonyl activity, for coating systems ranked from best to worst performance (1 = best, 4 = worst), compared against the calculated ranking based on the number of attack sites from the dual descriptor Fukui function, and the rankings from approximate and exact chemical hardness. Shaded rows indicate matches to computationally derived performance ranking from the number of attack sites

			HH*8020Br	HH*8515Br	CHDA*8020Br	CHDA*8515Br
Computational data	Reactive zones	Total	32.60	33.45	46.40	48.55
		Electrophilic	17.80	18.35	22.80	23.85
		Nucleophilic	14.80	15.10	23.60	24.70
	Calculated coating rank		1	2	3	4
Experimental data	Final% gloss retention	Accelerated	1	2	3	4
		Natural	1	2	3	4
	Δ*E*	Accelerated	2	1	3	4
		Natural	1	2	3	4
	% Change in hydroxyl activity	Accelerated	2	1	4	3
		Natural	1	2	3	4
	% Change in carbonyl activity	Accelerated	1	2	3	4
		Natural	1	2	3	4
Approximate chemical hardness rank			4	2	3	1
Exact chemical hardness rank			2	1	4	3

The ranking of coatings according to the number of reactive sites with a weighting based on the resin : HMMM ratios, is HH*8020Br, HH*8515Br, CHDA*8020Br, and CHDA*8515Br, from best to worst, with a greater number of reactive sites indicating a poorer performance. The accelerated and natural weathering tests show similar correlation, with the predicted performance ranking matching gloss and colour retention performance of samples. The most noticeable correlation between experimental and computational results is within naturally weathered samples, where the experimental performance of the coatings matches the predicted rankings across all investigated areas. The good agreement between natural weathering tests and DFT calculations highlights the potential of DFT for the predicting the formation of degradation products, when accelerated tests fail.

Comparing coating performance against the rankings identified through computational modelling showed similarities between the computational rankings and the performance of the coatings under experimental conditions. Further analysis would be needed to extrapolate these results to other coating systems. Looking at the coatings in terms of performance following weathering against the rankings identified through computational modelling showed significant correlation between computational and experimental results, with *R*^2^ values showing that 7 of the 8 sets of results provide values greater than 0.7, indicating statistical significance.

**Fig. 11:**
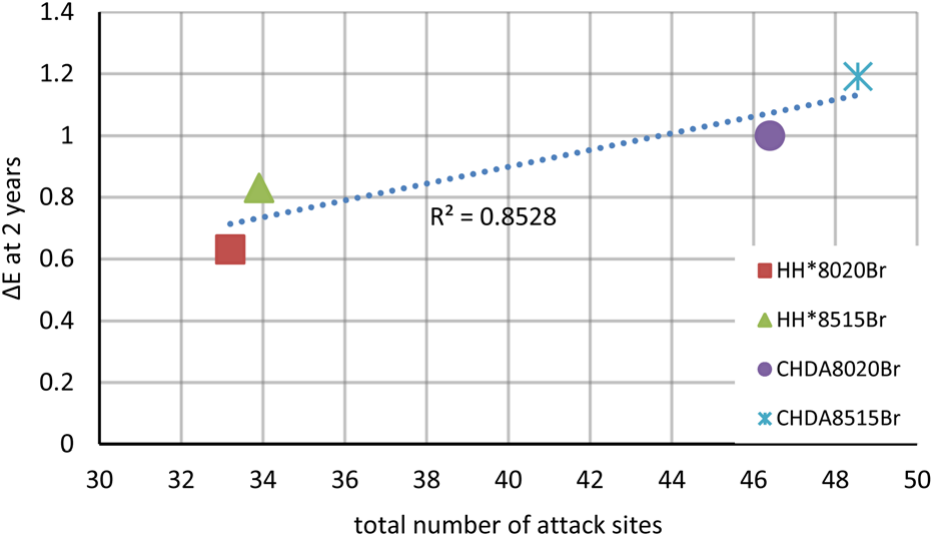
Linear correlation between the number of molecular sites that would undergo nucleophilic or electrophilic attacks according to the dual descriptor of the Fukui function against the Δ*E* of samples exposed in natural weathering tests.

Comparisons were made between the number of sites in the molecular structure that would undergo nucleophilic or electrophilic attacks according to the dual descriptor of the Fukui function against Δ*E*, percentage gloss retention, percentage change in hydroxyl activity, and the percentage change in carbonyl activity. Fig. 11 demonstrates an example, with the subsequent graphs included in the ESI,† with a summary of the results provided in Table 6.

**Table 6:** Highlighting the *R*^2^ linear correlation value and *y* = *mx* + *c* values for graphs comparing Δ*E*, percentage gloss retention, percentage change in normalised hydroxyl activity, and percentage change in normalised carbonyl activity for samples subjected to natural and accelerated weathering

	*R* ^2^ value	*y* = *mx* + *c*
Δ*E* after 2 years	0.8528	0.0273*x* − 0.1921
Δ*E* after 2000 hours	0.9371	0.0329*x* − 0.594
% Gloss retention at 2 years	0.9946	−3.9597*x* + 198.64
% Gloss retention at 2000 hours	0.9993	−4.6739*x* + 235.5
% Hydroxyl change at 2 years	0.5695	3.6038*x* + 93.003
% Hydroxyl change at 2000 hours	0.9505	5.9824*x* − 131.59
% Carbonyl change at 2 years	0.9991	4.6331*x* − 126.5
% Carbonyl change at 2000 hours	0.9883	4.267*x* − 135.93

Most of the graphs show a strong linear corelation, as represented by the high *R*^2^ values. The smallest *R*^2^ value (0.5695) corresponds to the percentage change in the normalised hydroxyl activity as identified through IR analysis. It is important to remember that the hydroxyl region can be influenced by post-curing reactions and NH activity it is possible that these factors were responsible for the differences observed in correlation between naturally weathered and accelerated samples. Naturally weathered samples are also exposed to additional factors that cannot be replicated in accelerated tests, such as biological fouling and pollution that also exert an influence over degradation. Table 6 highlights that the majority of the results produced by comparing the number of computationally identified attack sites to experimental weathering performance are statistically significant.

To aid further understanding, vibrational frequency data for each of the models has been investigated and included within an ESI.† The initial data for some of the models indicated the presence of imaginary modes. Models were then manually re-optimised, and the frequency calculations repeated until imaginary modes were confirmed to no longer be present.

## 4 Conclusions

This study has identified statistically significant correlations between the number of attack sites and the performance of coating systems investigated through accelerated and natural weathering. One of the most significant values gives an *R*^2^ value of 0.9991 for the relation between the percentage change in normalised carbonyl activity of naturally weathered samples and the number of attack sites. This is particularly important as the experimental data showed that samples from accelerated weathering did not match this pattern, indicating that the method of weighting computationally derived data laid out within this study provides additional information that would not be obtainable through accelerated weathering tests. Of 8 comparison plots, 7 were found to have a strong statistical significance (>0.7 *R*^2^ value), with 6 graphs demonstrating an *R*^2^ value greater than 0.9, highlighting that computational modelling can be reliably used to provide statistically relevant information on predicted percentage gloss retention, Δ*E*, and the average percentage change in carbonyl activity.

This study has highlighted a statistically significant relationship between the weighted rankings of computationally derived attack sites and the results of real-world weathering data and presents a framework that validates the use of computational modelling within the design and analysis of novel coating systems. With the use of high throughput computing these results can be supplied in hours or days rather than in the months and years that accelerated and natural weathering tests require, significantly reducing time and energy costs, along with reducing material wastage.

Further work in this area would be well spent investigating for the potential correlation between molecular volume or molecular surface areas and experimentally determined coating properties to further improve understanding within the area.

With further work that expands on experimentally validating computationally compiled data it can become possible to produce large data sets that can be used in ML training to provide rapid feedback on proposed resin systems, which would have the potential to streamline the development of novel coating systems.

## Author contributions

Conceptualisation: CB, methodology: CB, FMM, validation: CB, CG, FMM, formal analysis: CB, FMM, EJ, investigation: CB, FMM, resources: JS, FMM, EJ, IM, data curation: CB, FMM, writing – original draft: CB, writing – review and editing: CB, FMM, EJ, CG, IM, visualisation: CB, FMM, EJ, supervision: EJ, IM, JS, project administration: EJ, IM, funding acquisition: IM, EJ, JS, FMM.

## Conflicts of interest

There are no conflicts to declare.

## Supplementary Material

RA-014-D3RA06744K-s001

RA-014-D3RA06744K-s002

RA-014-D3RA06744K-s003

RA-014-D3RA06744K-s004

RA-014-D3RA06744K-s005

RA-014-D3RA06744K-s006
