# Supplementary material for: Computationally-accelerated prediction of polyester-melamine coatings degradation to design sustainable organically-coated steels for outdoor applications
Source: RSC Adv. 2024 Jun 24;14(26):18343–54. doi: 10.1039/d3ra06744k (PMC11194856; doi:10.1039/d3ra06744k)
Supplement: RA-014-D3RA06744K-s001 [file RA-014-D3RA06744K-s001.pdf]

Electronic Supplementary Material (ESI) for RSC Advances.
This journal is © The Royal Society of Chemistry 2024

Table 1 - Overview of the identified electrophilic and nucleophilic attack sites of computationally-produced model structures

Model	Number of electrophilic sites	Number of nucleophilic sites	Total
HHPA	8	8	16
HHPA NPG	8	8	16
HHPA TMP	9	3	12
HHPA HMMM	9	10	19
CHDA	8	12	20
CHDA NPG	12	10	22
CHDA TMP	7	6	13
CHDA HMMM	6	6	12
TMA	10	18	28
TMA NPG	8	8	16
TMA TMP	9	15	24
TMA HMMM	16	16	32
DDSA	5	8	13
DDSA NPG	4	7	11
DDSA TMP	6	7	13
DDSA HMMM	6	10	16

Table 1 gives an overview of the number of electrophilic and nucleophilic attack sites as identified through computational chemical modelling.

Table 2-Weighted attack sites as a function of the polyester-melamine ratio.

Monomer base	Number of electrophilic sites	Number of nucleophilic sites	Total
HHPA	17.8	14.8	32.6
CHDA	22.8	23.6	46.4
TMA	24.8	35.2	60.0
DDSA	13.2	19.6	32.8

Table 2 breaks down the number of electrophilic and nucleophilic attack sites for the monomers investigated within the manuscript. It also includes Dodecenyl succinic anhydride (DDSA) and Trimellitic anhydride (TMA) based coating systems that have been calculated. The two new

monomers have been included to show that other monomers of past and present interest to the coatings industry.

The results suggest the DDSA would produce a coating that is better able to resist weathering degradation than CHDA and is comparable to HHPA. Likewise, TMA is projected to produce a coating that weathers more rapidly than the coatings investigated within this study.

Further work would look at producing coating systems based on DDSA and TMA and approximately equivalent to those already investigated. The new coating systems would then be subjected to extended weathering programs as described within the manuscript so that a more accurate comparison could be made.
