# Supplementary material for: Computationally-accelerated prediction of polyester-melamine coatings degradation to design sustainable organically-coated steels for outdoor applications
Source: RSC Adv. 2024 Jun 24;14(26):18343–54. doi: 10.1039/d3ra06744k (PMC11194856; doi:10.1039/d3ra06744k)
Supplement: RA-014-D3RA06744K-s002 [file RA-014-D3RA06744K-s002.pdf]

Electronic Supplementary Material (ESI) for RSC Advances.
This journal is © The Royal Society of Chemistry 2024

HHPA

Mode	freq (cm**-1)	T**2	TX	TY	TZ
6:	99.57	0.268317	(-0.225872	-0.431086	0.177380)
7:	146.57	6.811966	(0.127422	1.008916	2.403709)
8:	168.10	1.175218	(0.787263	-0.083316	0.740603)
9:	220.63	2.204913	(1.249437	-0.763683	-0.246190)
10:	286.25	3.307129	(-0.778637	-1.498133	0.675611)
11:	311.02	1.988841	(-0.359598	-0.359523	-1.315399)
12:	326.61	5.876329	(-1.039807	-2.005734	0.878727)
13:	437.79	1.841176	(-0.574982	-1.125052	0.494802)
14:	462.67	7.521221	(-2.258712	1.428633	0.615183)
15:	480.79	1.514308	(-0.533243	-1.012274	0.453056)
16:	534.13	4.788529	(-1.217741	1.229925	1.338999)
17:	615.37	0.334102	(0.246019	0.481226	-0.204936)
18:	649.17	1.567861	(-0.533131	-1.037603	0.454984)
19:	659.34	7.088854	(-0.187965	-0.993267	-2.463117)
20:	748.82	23.970072	(4.428850	-1.910968	0.838787)
21:	803.45	0.551395	(0.322571	0.610940	-0.272206)
22:	837.85	2.846123	(-0.687979	-1.417315	0.603346)
23:	854.61	4.242180	(-1.749582	1.029040	0.349600)
24:	877.87	0.548659	(0.365161	0.581246	-0.278333)
25:	903.90	350.543077	(15.906017	-9.465489	-2.818902)
26:	928.68	0.303854	(-0.491558	0.184199	-0.168214)
27:	943.90	32.640293	(-4.984118	2.760124	0.424948)
28:	953.97	2.542778	(-0.753976	-1.278052	0.583850)
29:	1057.39	20.831928	(2.742987	-2.556714	-2.602146)
30:	1061.57	0.020646	(-0.012299	-0.143031	0.006053)
31:	1087.90	8.906398	(-1.894654	1.685887	1.573044)
32:	1114.52	9.923184	(-1.319639	-2.624178	1.138169)
33:	1142.40	13.114797	(1.543685	2.991532	-1.335131)
34:	1168.26	17.191606	(-2.981420	2.348702	1.669232)
35:	1199.08	128.511239	(4.851141	9.351091	-4.187453)
36:	1236.27	35.116517	(2.540932	4.887378	-2.184885)
37:	1250.37	4.244813	(1.835109	-0.920630	0.172132)
38:	1266.12	5.016343	(-0.961412	-1.852585	0.812378)
39:	1302.89	0.926931	(-0.660267	0.060157	-0.698111)
40:	1322.88	12.252748	(1.478318	2.905996	-1.273777)
41:	1329.40	0.220291	(-0.446571	0.100288	-0.103958)
42:	1359.26	0.715708	(-0.351584	-0.704199	0.310163)
43:	1373.27	0.781464	(0.365318	0.744118	-0.307073)
44:	1375.24	0.292181	(-0.375306	0.252532	0.295895)
45:	1387.86	6.469658	(-1.081811	-2.111855	0.916193)
46:	1397.41	3.470935	(1.366581	-1.012331	-0.760642)
47:	1492.27	5.848120	(-0.135322	1.028988	2.184260)
48:	1492.68	3.415201	(0.753696	1.527236	-0.717422)
49:	1499.31	10.573537	(-2.549001	0.422144	-1.974316)
50:	1507.05	0.083425	(-0.069748	-0.237786	0.148384)
51:	1852.51	625.839055	(21.947771	-11.922298	-1.411806)
52:	1915.45	138.226117	(4.981742	9.730004	-4.328440)
53:	2955.81	3.473410	(0.845756	1.573358	-0.531650)
54:	2969.73	7.272630	(-0.584024	-0.687046	-2.541557)
55:	2997.22	8.422967	(1.288697	2.026227	-1.629917)
56:	3005.14	25.067964	(-1.056804	2.770925	4.033994)
57:	3021.20	8.425455	(2.169159	1.074187	1.601976)
58:	3021.60	33.906854	(3.455478	-0.362101	4.672838)
59:	3055.17	42.507495	(3.879045	5.036214	-1.448121)
60:	3057.06	41.001270	(-4.351347	2.160761	-4.171110)
61:	3074.01	9.355380	(-0.572993	2.227027	2.016782)
62:	3074.96	36.573101	(-2.194024	2.783684	4.900047)

The first frequency considered to be a vibration is 6
The total number of vibrations considered is 57

HMMM and HHPA

Mode	freq (cm**-1)	T**2	TX	TY	TZ
6:	4.52	0.000000	(0.000000	0.000000	0.000000)
7:	12.70	0.472164	(0.575164	-0.345000	-0.149417)
8:	13.21	0.230649	(0.198045	-0.314011	0.304671)
9:	18.95	0.132739	(0.260951	-0.243504	0.073143)
10:	24.39	0.956683	(-0.368376	-0.562969	0.709964)
11:	32.38	1.084810	(-0.075528	1.032138	-0.117458)
12:	35.53	1.581257	(-0.290418	-1.065114	0.602035)
13:	35.90	1.472903	(-1.047881	-0.571837	-0.218749)
14:	50.26	0.106253	(-0.282338	0.151398	0.060143)
15:	55.60	2.031120	(-0.322149	0.818943	-1.121014)
16:	69.12	1.121920	(-0.893828	0.138770	-0.551120)
17:	74.78	2.136226	(-0.494155	1.290554	0.475928)
18:	79.52	2.985970	(1.513419	-0.787193	-0.275427)
19:	91.86	1.850928	(0.351794	-0.756198	-1.074864)
20:	97.15	5.663375	(1.971731	1.325488	-0.136868)
21:	119.37	1.772790	(-0.964685	0.714649	0.575717)
22:	127.48	1.128221	(0.213160	-0.841271	-0.612410)
23:	134.52	0.577160	(-0.734799	-0.188547	-0.040994)
24:	140.75	2.150962	(0.587328	1.255078	-0.480402)
25:	158.85	0.519282	(0.206613	-0.088045	-0.684720)
26:	160.50	0.533723	(0.569056	-0.293917	-0.351440)
27:	164.05	0.854303	(0.836811	0.258692	0.295176)
28:	172.84	1.819159	(1.295489	-0.343959	-0.150200)
29:	176.41	1.430456	(-0.867689	-0.813795	-0.123731)
30:	186.07	0.818606	(-0.858190	-0.204036	0.201208)
31:	200.48	1.408471	(-0.467342	-1.035123	-0.344358)
32:	208.67	11.855768	(-1.702637	-2.274928	1.944608)
33:	229.65	5.977844	(-0.596656	2.356354	0.263515)
34:	238.70	11.977967	(2.751346	1.512650	-1.456006)
35:	252.24	3.353510	(1.005420	1.355142	-0.711499)
36:	272.07	0.868226	(-0.583200	0.196462	0.699648)
37:	293.78	3.670692	(-0.531425	-1.840288	-0.040229)
38:	297.79	0.773910	(0.747090	0.281513	-0.369482)
39:	307.01	0.821982	(-0.405302	0.349540	-0.731802)
40:	313.92	5.532396	(-0.205866	-2.314854	0.362580)
41:	320.47	1.087640	(0.467937	-0.610450	0.704291)
42:	349.67	1.678152	(-0.659079	-0.670319	0.891313)
43:	376.18	5.124588	(1.945691	1.156615	0.033405)
44:	379.42	5.750431	(1.786538	1.599064	-0.041310)
45:	406.04	6.286681	(-2.249044	-1.017346	-0.439877)
46:	412.79	2.665538	(0.972214	0.760873	1.068368)
47:	423.88	2.367713	(-1.371237	-0.528559	0.456124)
48:	448.06	2.879805	(-0.164690	-1.680342	-0.170682)
49:	488.63	39.168178	(6.216024	0.687567	0.237640)
50:	494.94	11.115580	(-2.264548	1.711078	1.749176)
51:	511.13	30.871822	(-2.467982	4.958332	0.442529)
52:	537.98	54.493270	(7.011385	-0.900993	-2.126490)
53:	548.59	16.802501	(3.464617	-2.167432	0.318070)
54:	561.12	14.937617	(3.620907	0.664480	1.176909)
55:	567.15	36.913262	(2.287081	5.611018	-0.446097)
56:	608.46	1.342914	(0.388641	1.084541	0.125068)
57:	629.46	8.991988	(-2.520696	1.622935	-0.064514)
58:	645.36	24.783579	(-3.978015	2.961216	0.436091)
59:	656.51	51.117438	(0.250996	-7.072781	-1.014988)
60:	715.35	4.315711	(-0.926634	1.759263	0.601710)
61:	723.77	56.609858	(-6.233707	-4.207643	-0.215620)
62:	739.08	22.072524	(-3.835730	-2.364758	1.329519)
63:	757.18	74.432146	(-0.343686	-8.587214	0.757490)
64:	757.78	7.512912	(2.476808	-1.107821	0.388675)
65:	786.47	53.731587	(0.428949	7.219937	1.191678)
66:	799.25	1.727741	(-0.083174	-1.230373	-0.454978)
67:	823.06	58.817794	(7.122190	-2.843214	-0.091306)
68:	836.01	100.847691	(9.814863	-1.410504	1.589540)
69:	850.12	20.813110	(-3.289864	-3.014848	0.948997)
70:	855.70	40.676234	(-4.151970	-0.009589	4.841207)
71:	906.57	12.412813	(-1.391417	-3.183493	-0.584929)
72:	911.44	4.882154	(0.410694	0.154204	-2.165573)
73:	924.43	6.587778	(-0.864846	-2.316549	0.688053)
74:	939.57	77.654236	(-3.182213	-8.173839	0.846234)
75:	953.61	6.525066	(1.577191	-0.735023	1.870100)
76:	972.55	57.217122	(-3.675161	-6.446429	1.467605)
77:	973.88	24.314258	(2.846606	-3.494633	-1.999658)

78:	991.22	48.946149	(4.565785	2.053234	-4.887124)
79:	1000.62	18.267483	(0.481346	4.178568	0.758524)
80:	1011.43	245.409032	(5.563172	14.605892	1.062101)
81:	1015.89	23.978409	(4.286690	0.388828	-2.334847)
82:	1026.25	121.413756	(-8.700424	-6.685312	-1.011428)
83:	1041.15	26.646456	(4.112138	-1.783495	-2.560453)
84:	1043.20	59.287079	(-7.382590	1.332561	1.734570)
85:	1051.88	46.201608	(4.376740	-3.409358	-3.927090)
86:	1073.29	13.724717	(2.798626	2.367277	-0.537039)
87:	1097.60	76.004284	(6.319525	5.787927	1.602431)
88:	1100.74	125.558300	(2.832186	1.071670	10.788352)
89:	1104.45	76.609373	(0.551531	4.607381	7.421403)
90:	1107.27	104.702635	(3.698733	4.647630	-8.331959)
91:	1123.69	215.801058	(-12.846403	-1.468749	-6.972357)
92:	1130.87	194.965376	(-7.169805	-11.292262	4.005509)
93:	1143.86	117.022167	(1.521461	-9.118540	5.617789)
94:	1165.25	8.115957	(-1.779509	-1.618652	1.526195)
95:	1167.89	2.705436	(0.085062	1.631269	-0.192776)
96:	1171.00	5.469305	(-2.113276	-0.988492	0.162023)
97:	1172.61	3.949457	(-1.560383	-0.603111	-1.072809)
98:	1176.81	0.602243	(-0.684240	0.073071	0.358774)
99:	1177.75	6.211630	(-2.375044	0.253889	0.711573)
100:	1183.51	57.889795	(-6.737480	-0.194960	-3.529610)
101:	1206.13	3.477650	(-1.693813	-0.771116	0.118435)
102:	1210.52	30.888027	(0.618680	-0.105970	5.522140)
103:	1211.68	5.571138	(1.763783	-0.996167	-1.211552)
104:	1216.29	7.396528	(-1.527456	-1.557444	-1.624122)
105:	1217.02	3.923891	(1.112888	-0.177094	1.629113)
106:	1238.08	68.389870	(2.577724	-7.685330	1.637351)
107:	1245.79	13.229650	(2.960145	-2.066968	-0.441400)
108:	1247.68	16.870044	(2.796956	3.006677	0.083514)
109:	1299.08	8.865567	(-1.244273	-2.699607	-0.171682)
110:	1306.62	1.616501	(0.907367	0.183750	0.871449)
111:	1319.20	5.610746	(0.640598	1.909144	-1.247216)
112:	1334.61	44.864283	(5.194011	4.054347	1.203660)
113:	1340.78	91.070302	(-7.859819	-5.367859	0.692560)
114:	1344.48	98.551617	(8.020997	5.666709	-1.450391)
115:	1365.16	6.905671	(2.536369	-0.096196	-0.680624)
116:	1373.22	0.756453	(0.260792	0.589476	-0.583916)
117:	1381.48	11.701289	(0.826299	-2.922775	1.573501)
118:	1385.29	55.302161	(-5.315573	4.174686	-3.101427)
119:	1393.77	381.674379	(-18.371167	5.757949	3.319730)
120:	1401.63	16.255961	(-2.434997	3.052288	-1.005130)
121:	1406.95	16.077725	(3.130987	2.419852	0.647270)
122:	1424.65	56.646784	(-5.643137	-4.958248	0.466443)
123:	1449.67	150.251796	(-1.176199	-12.201145	-0.020402)
124:	1457.42	3.284022	(-0.967949	-1.106589	1.059508)
125:	1458.23	7.844288	(0.447903	2.734888	-0.405043)
126:	1462.54	1.589140	(0.900915	-0.543633	0.694229)
127:	1473.97	4.727517	(1.223706	-1.643011	0.728405)
128:	1476.72	9.060514	(2.862797	0.853910	0.368435)
129:	1479.43	3.786786	(0.021097	1.307092	1.441475)
130:	1480.85	1.810246	(0.750369	1.116565	0.021821)
131:	1481.97	9.944508	(-0.641847	-2.883126	1.104593)
132:	1483.36	5.309347	(-1.261353	1.426470	-1.297505)
133:	1485.45	4.472347	(1.773297	0.284356	-1.116650)
134:	1494.10	4.107384	(-1.188174	-1.609166	0.325902)
135:	1495.84	6.407449	(2.029513	-1.378952	0.622107)
136:	1497.24	9.139231	(-1.743939	0.608918	2.393142)
137:	1500.70	11.651902	(3.168493	0.699543	1.059809)
138:	1504.76	3.857731	(-0.066306	1.401536	1.374420)
139:	1506.37	1.787794	(0.718322	0.721851	-0.866451)
140:	1508.91	7.254529	(0.126633	2.099027	1.683027)
141:	1509.69	1.916725	(0.682253	-0.696974	0.982590)
142:	1510.22	9.911663	(-1.410449	0.669422	-2.733893)
143:	1581.80	359.494636	(-0.570532	-18.943257	0.567583)
144:	1589.04	668.571668	(25.744239	-0.281531	-2.393022)
145:	1800.64	229.558462	(-0.337725	-2.777079	14.890676)
146:	1831.91	198.928959	(6.403419	-1.560439	-12.469571)
147:	2941.52	31.896574	(-5.614627	-0.583957	0.177566)
148:	2979.28	48.666199	(4.209798	0.157025	5.560498)
149:	2987.55	72.636962	(-5.472929	-6.287388	1.775602)
150:	2997.49	16.879946	(3.407741	2.257871	-0.411417)
151:	3007.17	9.653907	(-2.020670	1.594489	-1.740232)
152:	3012.64	4.421389	(-0.659023	1.743207	0.973812)
153:	3014.23	19.568531	(-2.544192	-3.285087	-1.517835)
154:	3018.30	65.667273	(5.241777	-4.180427	-4.551382)
155:	3019.22	42.072986	(3.102659	-4.287270	-3.750441)
156:	3026.68	48.123531	(-3.469460	-5.591547	2.195674)

157:	3027.07	30.426788	(-4.520665	1.958749	2.480661)
158:	3035.95	39.429483	(4.289508	-4.021373	-2.204123)
159:	3036.62	16.947867	(1.888323	0.782369	3.573514)
160:	3065.23	35.335471	(-3.815346	4.558224	0.034734)
161:	3067.66	24.690122	(1.699866	2.384042	-4.014588)
162:	3077.76	15.449131	(-2.734582	2.279150	-1.666333)
163:	3081.32	32.199816	(-4.954796	-2.304820	1.528926)
164:	3083.86	12.716325	(1.577336	1.262621	-2.938388)
165:	3084.54	24.743034	(4.763985	0.408411	1.371380)
166:	3084.96	27.775204	(-2.493875	4.308655	-1.729531)
167:	3111.53	23.891482	(-2.042127	3.284298	2.989078)
168:	3114.22	13.864994	(3.131843	1.735558	1.021956)
169:	3115.86	14.691591	(-0.204941	-2.947348	2.441870)
170:	3117.28	19.698463	(1.111278	-0.410950	4.277224)
171:	3121.67	16.602374	(2.884534	1.063061	2.674274)
172:	3130.48	8.608174	(1.284477	-1.357467	2.261763)
173:	3743.62	73.235728	(8.335545	0.943215	-1.692564)

The first frequency considered to be a vibration is 6
The total number of vibrations considered is 168

CHDA

Mode	freq (cm**-1)	T**2	TX	TY	TZ
6:	15.06	1.256134	(0.744146	0.585430	-0.599710)
7:	23.26	0.669801	(0.669859	0.357347	0.305604)
8:	53.63	0.368652	(-0.081625	0.042550	-0.600149)
9:	130.90	0.539515	(0.670293	-0.248329	-0.168982)
10:	169.00	1.201928	(0.607300	-0.906065	-0.110273)
11:	213.24	0.463360	(-0.154321	0.537390	0.388275)
12:	220.59	0.099455	(0.158980	0.058318	0.266043)
13:	250.61	0.515127	(-0.396178	0.050654	-0.596326)
14:	295.89	2.067343	(0.318229	-0.391275	-1.346468)
15:	359.42	0.270333	(-0.109701	-0.477792	-0.173245)
16:	437.33	16.935883	(3.231724	-2.537329	0.231956)
17:	457.43	1.575560	(0.771009	-0.986641	0.087431)
18:	498.79	6.055845	(-1.882413	-0.285338	-1.559150)
19:	518.72	1.601595	(-0.130171	1.215992	-0.325598)
20:	592.40	127.527496	(8.192437	7.176683	-2.984407)
21:	600.11	19.729881	(-1.176810	1.509485	-4.008298)
22:	634.18	18.806419	(1.097990	2.358918	3.469344)
23:	663.12	68.591706	(6.318751	-4.149791	-3.382946)
24:	741.38	43.670193	(3.010291	4.907426	-3.244304)
25:	771.13	17.248144	(3.755962	1.491708	0.956924)
26:	793.75	4.429824	(-0.022286	-1.147085	1.764518)
27:	803.66	1.376855	(1.016018	-0.145625	0.568643)
28:	872.95	9.545867	(-1.430529	0.035107	-2.738288)
29:	910.66	10.588559	(1.018634	-2.781324	1.347287)
30:	919.02	3.608628	(1.695913	-0.069804	-0.853015)
31:	923.01	7.511628	(0.608865	1.396227	2.278478)
32:	984.56	5.824620	(1.373566	-1.422527	1.383601)
33:	1020.72	41.918629	(-4.107515	4.192304	2.733410)
34:	1041.52	2.546074	(0.949004	-1.273883	-0.150627)
35:	1073.16	3.209277	(1.527077	-0.540693	0.764829)
36:	1085.10	4.260010	(1.104447	-1.114956	1.340551)
37:	1110.34	89.408524	(8.291018	-4.545746	-0.061190)
38:	1129.68	162.699066	(7.618836	-2.537622	9.910241)
39:	1161.14	102.325912	(-4.450231	0.428013	-9.074038)
40:	1190.20	140.981300	(-6.109290	6.427072	7.896241)
41:	1245.46	2.158733	(1.381593	0.497372	-0.050564)
42:	1262.85	17.385008	(1.944474	-0.695702	3.622158)
43:	1277.62	2.275257	(-0.370825	1.003748	1.063126)
44:	1303.45	2.155663	(0.761462	-0.396929	1.190918)
45:	1324.56	2.742542	(-0.551026	1.489577	0.469119)
46:	1332.51	7.003155	(-1.768706	1.967931	0.045630)
47:	1343.80	0.265100	(-0.174306	0.458786	-0.155669)
48:	1350.10	49.254227	(3.015570	-4.558359	-4.402491)
49:	1361.48	16.126252	(-2.612978	2.859600	-1.058907)
50:	1370.84	0.574689	(0.679719	-0.126505	0.310914)
51:	1373.91	9.913220	(2.499892	-0.822077	1.728569)
52:	1398.69	3.516613	(1.585453	-0.969273	0.251915)
53:	1413.28	31.953379	(-3.662469	3.747494	-2.120375)
54:	1490.91	0.350857	(-0.233170	-0.516495	-0.172401)
55:	1494.01	5.354559	(-1.676454	-1.522362	-0.475891)
56:	1500.72	17.695029	(-1.755648	2.088670	3.201591)

57:	1508.18	0.200989	(-0.200271	0.388132	-0.101162)
58:	1804.86	251.866273	(2.893824	-15.010112	4.264808)
59:	1806.67	390.075362	(7.279251	2.279443	18.217903)
60:	3018.31	7.021959	(-2.455250	0.188614	0.978842)
61:	3029.71	16.228107	(-2.661754	-1.809594	2.422508)
62:	3030.41	9.598147	(3.070830	0.288615	-0.291295)
63:	3032.54	18.280818	(-0.176119	3.191788	2.839417)
64:	3040.94	15.210959	(-0.762911	-3.309395	-1.917507)
65:	3049.66	2.858730	(1.211057	-1.014312	-0.602695)
66:	3066.46	41.216343	(-5.482752	-3.336787	0.147062)
67:	3072.77	3.836888	(-0.855882	-0.123156	1.757608)
68:	3077.69	8.134086	(-2.516586	-1.325054	0.212395)
69:	3083.34	62.480766	(1.129482	-1.445642	7.688638)
70:	3745.94	70.154142	(-5.551985	6.263320	0.316893)
71:	3750.07	69.819946	(-4.105634	6.619351	3.024550)

The first frequency considered to be a vibration is 6
The total number of vibrations considered is 66

HMMM and CHDA

Mode	freq (cm**-1)	T**2	TX	TY	TZ
6:	8.95	0.010529	(-0.046963	0.019642	0.089093)
7:	16.89	0.460348	(0.488018	-0.003467	-0.471354)
8:	17.91	0.132414	(0.061150	0.230115	0.275177)
9:	20.40	1.293623	(0.844109	-0.369286	0.666881)
10:	29.96	0.322325	(0.206470	0.479475	-0.223156)
11:	33.52	0.452213	(-0.671051	-0.010685	-0.042301)
12:	42.85	0.106923	(-0.030740	-0.321548	0.050845)
13:	53.08	2.419931	(0.941170	-0.286798	1.204939)
14:	60.16	2.599109	(1.230813	1.028282	-0.163840)
15:	69.42	1.938002	(0.322417	1.304719	-0.362986)
16:	74.58	0.536742	(-0.529469	0.270651	-0.427963)
17:	78.11	2.361456	(-0.514205	-0.849445	-1.172813)
18:	86.46	1.792063	(0.699454	1.106169	-0.281455)
19:	96.09	6.808624	(2.242757	-0.771106	-1.088145)
20:	99.94	3.094298	(0.622315	1.581774	-0.452783)
21:	116.55	0.346836	(0.494430	0.023166	-0.319122)
22:	126.96	2.092361	(0.706872	-1.131459	-0.559012)
23:	137.41	1.220543	(0.341457	0.965271	-0.414973)
24:	137.88	0.138318	(0.139733	0.336364	-0.075179)
25:	158.71	0.829131	(-0.901242	-0.121371	0.046502)
26:	160.64	1.616540	(-0.603847	-0.813854	-0.767822)
27:	164.53	1.527987	(-1.214459	0.188509	0.132443)
28:	169.15	1.182402	(-0.424182	0.792443	0.611969)
29:	179.71	1.203903	(-0.643436	-0.602315	0.653537)
30:	186.13	1.207233	(0.680542	0.554100	0.661111)
31:	197.94	4.641366	(1.510966	1.430951	-0.557431)
32:	201.89	2.469876	(0.688470	0.752049	-1.195955)
33:	210.16	3.706260	(-0.441137	-1.530693	1.081035)
34:	238.52	5.260832	(-1.277009	1.489915	1.187532)
35:	250.99	10.869423	(-2.573664	-1.410842	1.501733)
36:	256.21	1.371197	(-0.996495	0.208675	-0.578489)
37:	265.72	1.077807	(-0.013530	1.037903	-0.019516)
38:	282.04	0.529636	(-0.043849	-0.136083	0.713579)
39:	293.43	2.117332	(-0.468647	0.930606	-1.015713)
40:	311.67	5.567091	(0.035711	2.266806	-0.653761)
41:	323.96	0.496293	(0.357740	-0.141240	0.590225)
42:	330.37	1.214942	(-0.178977	0.571961	0.925078)
43:	361.27	1.729823	(0.224579	0.871720	-0.958901)
44:	382.16	5.686446	(1.298361	1.979491	-0.286918)
45:	399.19	2.856353	(0.400345	1.572590	-0.472270)
46:	432.75	4.974253	(-2.104444	-0.629261	0.386779)
47:	436.11	18.033386	(1.423678	3.931783	-0.740008)
48:	457.71	7.533956	(0.188350	2.635357	0.743890)
49:	486.44	0.707370	(-0.145856	-0.827611	0.034009)
50:	493.14	21.308233	(4.274289	-0.205489	-1.731028)
51:	510.55	1.467199	(-0.643436	0.989017	0.273922)
52:	515.33	85.554258	(8.309786	-3.221772	-2.474247)
53:	540.62	14.578756	(3.344972	0.965209	-1.567893)
54:	560.87	18.720503	(-3.578623	-2.289286	-0.820446)
55:	602.88	52.714211	(5.266687	-3.943651	3.069827)
56:	611.10	2.521826	(-0.735038	-0.330105	-1.368421)
57:	621.91	24.024924	(4.112755	-2.201714	1.504204)
58:	640.59	18.205268	(-3.900522	1.477539	0.898930)
59:	652.24	42.193758	(3.839680	5.044145	1.416765)

60:	718.57	10.099635	(-3.036943	-0.738292	-0.575793)
61:	735.04	36.174439	(4.165795	3.325947	-2.785438)
62:	745.72	36.311657	(-3.483499	4.411623	-2.171283)
63:	756.72	10.023814	(1.959988	2.457032	-0.381124)
64:	768.24	53.567378	(0.528594	7.085864	-1.754566)
65:	777.06	27.072270	(-2.251299	4.631339	0.744728)
66:	783.30	15.702827	(1.970440	3.262011	1.086037)
67:	814.81	69.438042	(-6.262817	5.486098	0.343355)
68:	838.96	126.081570	(-10.967408	-2.344416	0.548862)
69:	854.20	56.210451	(-4.531328	-1.057970	5.878624)
70:	887.21	10.591499	(1.082046	2.707382	-1.445946)
71:	899.79	16.669313	(-1.052161	-3.914208	-0.491167)
72:	911.74	4.379524	(1.632398	0.061588	-1.308055)
73:	932.47	83.194339	(2.226468	8.753426	-1.270715)
74:	947.75	13.248063	(0.406799	-3.596656	0.382941)
75:	955.68	139.642845	(-2.875815	-10.849174	3.697020)
76:	968.64	3.508107	(-0.855479	-0.106152	1.662827)
77:	970.12	43.296928	(0.809886	6.144964	-2.209169)
78:	981.73	9.837167	(-2.368725	-1.416676	1.489744)
79:	989.84	45.441574	(2.816519	-3.262354	-5.183227)
80:	1001.52	14.938304	(0.197672	3.841214	0.379877)
81:	1012.71	28.001873	(3.361522	3.821530	-1.448429)
82:	1024.82	96.010048	(-4.107406	-8.323389	3.140137)
83:	1029.06	85.187581	(4.956533	-7.078827	-3.242001)
84:	1045.59	3.912029	(0.375334	-1.517631	-1.211590)
85:	1050.49	60.848252	(5.635076	-1.838157	-5.071031)
86:	1079.80	5.774725	(-1.185121	-2.090502	0.004120)
87:	1099.33	100.790596	(-3.819386	-8.998472	2.287004)
88:	1100.31	173.474040	(-4.042223	-3.788488	-11.949135)
89:	1106.94	73.756951	(0.457525	-3.454408	-7.849502)
90:	1121.81	34.852204	(1.869819	5.592790	-0.276908)
91:	1132.65	0.556677	(-0.002614	-0.636371	0.389490)
92:	1142.68	301.920998	(-3.507933	-14.112994	9.509932)
93:	1154.19	162.818521	(-8.042374	-8.671312	4.790312)
94:	1160.56	97.049932	(6.018406	6.200344	-4.731222)
95:	1169.46	7.325383	(2.087942	1.435946	-0.950757)
96:	1170.35	2.206454	(1.096927	-0.913434	0.410904)
97:	1172.57	3.758418	(1.555971	0.493593	1.045820)
98:	1174.25	1.429572	(-0.794514	-0.798657	-0.400584)
99:	1175.40	1.695204	(-1.300070	0.059939	0.037791)
100:	1184.22	37.560201	(3.657373	-0.027053	-4.917631)
101:	1210.18	31.566030	(-0.179412	-0.915466	-5.540376)
102:	1213.00	3.791524	(1.516378	-0.327079	1.176921)
103:	1215.77	5.045618	(-0.671677	-1.368698	-1.649586)
104:	1216.96	3.636852	(-0.336597	1.306587	-1.347734)
105:	1237.48	69.832898	(2.136011	-7.751853	2.275771)
106:	1245.34	21.469813	(3.656013	-2.809088	-0.460879)
107:	1255.26	40.538078	(3.027342	4.537166	-3.284419)
108:	1270.35	9.785641	(-0.500237	-3.084404	0.147845)
109:	1278.43	18.031978	(-2.789671	-2.378709	2.142769)
110:	1284.19	86.447172	(-2.726495	-8.522299	-2.526622)
111:	1307.45	21.132876	(0.264776	-3.382911	-3.101400)
112:	1323.58	3.416192	(0.601533	0.445331	-1.689979)
113:	1343.82	132.514305	(9.447781	5.835193	-3.033852)
114:	1347.08	2.520854	(-1.340236	-0.124557	0.842085)
115:	1357.33	19.593519	(-1.967921	-2.682920	2.919374)
116:	1357.90	43.293533	(-0.451813	-5.821588	-3.032904)
117:	1372.54	2.143786	(0.650800	-0.706100	-1.105291)
118:	1381.07	51.821779	(-0.426934	4.258778	5.788118)
119:	1382.49	44.650387	(3.824654	-5.140525	1.896683)
120:	1392.74	399.735324	(-17.602740	7.534936	5.753573)
121:	1406.35	23.630081	(0.585029	1.143998	-4.688186)
122:	1413.57	36.336028	(-1.748713	-5.761304	0.292248)
123:	1424.69	30.696803	(-3.098109	-4.583265	0.303653)
124:	1447.55	121.692943	(1.718472	10.825902	-1.240828)
125:	1457.47	1.568735	(0.737288	1.001848	-0.146433)
126:	1458.71	13.745913	(0.884752	-3.600314	-0.029434)
127:	1461.42	2.102279	(-1.262873	0.094045	-0.706105)
128:	1473.79	4.222316	(-0.730815	1.803281	-0.660607)
129:	1476.40	2.132841	(-1.360357	-0.018090	-0.530984)
130:	1478.86	4.331388	(-0.176039	-1.542993	-1.385486)
131:	1481.65	2.687571	(1.319893	0.943282	-0.235946)
132:	1481.91	10.288022	(3.046081	0.872976	0.497316)
133:	1485.56	4.511734	(1.817690	0.031909	-1.098507)
134:	1493.20	4.882109	(-1.451823	-1.595187	0.479266)
135:	1494.87	3.264905	(-1.046961	-1.407038	0.434767)
136:	1496.15	4.093974	(0.080003	1.685506	1.116531)
137:	1503.01	3.688206	(1.680843	-0.568727	0.734523)
138:	1504.33	9.200930	(0.590598	-2.294467	1.894082)

139:	1505.35	4.111813	(0.035482	-1.915267	-0.665061)
140:	1508.65	5.152198	(-1.219145	-1.901144	-0.227010)
141:	1508.90	3.945727	(-0.994087	1.466624	-0.898072)
142:	1510.37	8.346785	(-1.253268	0.523238	-2.549966)
143:	1519.23	6.264780	(-0.303913	-1.108392	2.223484)
144:	1581.47	363.224652	(-1.087550	18.967045	-1.514292)
145:	1589.66	621.502478	(-24.388646	-0.439324	5.148147)
146:	1803.18	278.447267	(-8.372805	2.624169	14.193560)
147:	2936.36	32.882401	(-5.731816	-0.052133	0.161161)
148:	2979.26	49.512978	(-4.443447	-0.606318	-5.422281)
149:	2988.30	69.961083	(5.589440	5.746081	-2.387844)
150:	3016.23	16.846812	(1.402225	3.486175	-1.651413)
151:	3019.79	76.026447	(5.505191	-4.312986	-5.207443)
152:	3020.17	3.238209	(-0.631764	1.059631	-1.310063)
153:	3026.72	49.178987	(-3.741154	-5.159872	2.925488)
154:	3030.58	29.487312	(-4.275512	-2.664626	-2.026592)
155:	3030.91	2.779929	(-0.887540	0.580248	-1.286668)
156:	3035.13	41.967057	(3.645201	-4.609566	-2.726072)
157:	3039.31	19.092071	(-3.593302	0.536951	2.427331)
158:	3045.22	1.657873	(0.493833	1.042607	0.571815)
159:	3048.23	19.451693	(2.884459	-2.122774	2.573989)
160:	3063.59	35.107559	(-3.585472	4.710907	-0.243528)
161:	3064.74	3.531258	(0.443632	0.183175	-1.816837)
162:	3069.25	17.542182	(3.905292	-1.427089	0.504276)
163:	3083.60	5.944602	(-2.002877	1.372026	0.225006)
164:	3084.48	37.433189	(-5.195559	-3.046729	-1.075547)
165:	3087.68	22.945167	(2.762910	-3.882025	0.491303)
166:	3091.14	37.816584	(-0.693746	-2.342786	5.643284)
167:	3109.40	23.111393	(1.821424	-3.635160	-2.565037)
168:	3113.05	15.264424	(-3.505247	-1.630211	-0.565756)
169:	3117.64	14.445723	(3.284266	0.750197	1.759695)
170:	3118.90	19.760469	(1.264376	-0.126134	4.259802)
171:	3122.83	14.864901	(0.237491	2.406985	-3.002486)
172:	3130.83	8.470911	(1.501759	-1.312989	2.119361)
173:	3739.63	73.151841	(1.863341	7.847489	2.845473)

The first frequency considered to be a vibration is 6
The total number of vibrations considered is 168

DDSA

Mode	freq (cm**-1)	T**2	TX	TY	TZ
6:	20.38	0.722560	(0.733580	0.372671	0.213393)
7:	27.37	0.860895	(-0.350914	-0.832932	-0.209710)
8:	58.23	0.929235	(-0.137936	-0.354103	0.885901)
9:	63.07	0.891133	(0.395052	0.714781	-0.473451)
10:	71.97	0.654951	(0.114163	-0.797417	0.077745)
11:	94.03	1.250053	(-0.028203	-0.632820	-0.921301)
12:	105.98	0.201852	(-0.400897	-0.178644	0.096024)
13:	123.81	2.006760	(0.429612	-1.017577	-0.886978)
14:	159.43	1.883089	(1.119237	-0.109636	-0.786370)
15:	169.83	0.204636	(-0.098423	-0.431560	-0.093299)
16:	202.15	0.124954	(-0.192455	-0.083596	-0.284477)
17:	206.50	3.481066	(1.414350	-0.610306	1.052713)
18:	219.76	0.984025	(-0.485020	0.264546	-0.823890)
19:	227.75	0.473019	(-0.269531	-0.177832	0.607247)
20:	250.20	1.670119	(1.189084	0.287922	0.416292)
21:	252.01	0.064084	(-0.239423	-0.070547	0.042237)
22:	253.31	0.087341	(-0.208237	-0.070416	-0.197535)
23:	280.62	1.298558	(0.861955	-0.018763	0.745145)
24:	284.81	2.632508	(-1.583228	-0.353492	0.030659)
25:	325.67	1.460255	(0.279479	0.194899	-1.159379)
26:	357.16	1.140909	(0.964867	0.342486	-0.304374)
27:	397.24	9.018744	(2.742889	1.002830	-0.699741)
28:	420.03	3.368334	(1.508768	1.044501	-0.031151)
29:	451.60	0.005045	(-0.038092	-0.027714	0.053164)
30:	457.81	10.867742	(-3.135658	-0.852255	0.555923)
31:	479.81	3.416187	(-1.507202	-0.861660	-0.634091)
32:	498.31	0.067922	(0.002732	0.126001	-0.228119)
33:	535.64	15.207689	(-2.778352	0.435667	2.701601)
34:	555.65	4.224830	(2.055228	0.019603	-0.021979)
35:	588.57	14.526663	(2.874568	-2.413999	-0.660405)
36:	603.54	12.847773	(-2.971026	1.067625	1.697337)
37:	658.74	62.401955	(7.319419	-1.905729	-2.279530)
38:	678.57	2.511778	(-0.994935	-0.109851	1.228745)

39:	749.63	23.547449	(4.696380	-0.776771	0.942385)
40:	812.81	10.292390	(-2.407197	-0.104613	2.118218)
41:	819.16	7.716320	(2.468510	-1.062460	-0.702820)
42:	824.24	2.133874	(-0.639909	-0.489915	1.218349)
43:	834.45	6.661600	(2.271044	-1.172385	-0.359822)
44:	846.53	42.755464	(4.070804	-4.964510	-1.240024)
45:	851.99	14.527618	(-0.259967	3.786235	0.352791)
46:	883.65	85.995497	(-6.854092	5.325544	3.264277)
47:	890.03	98.592150	(8.692302	-4.180670	-2.357548)
48:	901.93	34.883157	(5.544741	-1.575475	-1.287199)
49:	922.71	0.282514	(-0.048397	-0.505584	-0.156704)
50:	933.97	5.093243	(-2.195468	0.357785	-0.380988)
51:	969.04	14.674995	(3.187011	0.171857	2.118590)
52:	987.75	17.659106	(2.986207	1.133450	2.730744)
53:	997.61	0.665512	(0.236516	0.002880	0.780746)
54:	1009.51	9.524652	(2.716975	1.456763	0.143323)
55:	1014.85	0.262088	(-0.210376	0.247500	-0.395694)
56:	1018.37	12.903219	(-0.431251	-3.155738	-1.660892)
57:	1027.47	0.013420	(0.051735	0.102475	0.015575)
58:	1036.40	0.789092	(-0.105070	-0.270751	0.839491)
59:	1047.89	0.102922	(-0.301821	-0.108291	0.009947)
60:	1053.76	1.478268	(-0.969027	-0.705857	-0.202531)
61:	1062.44	13.150736	(-3.546085	-0.654043	0.385024)
62:	1068.20	0.840087	(-0.599836	-0.684223	-0.110101)
63:	1085.50	10.654625	(3.031467	1.174164	-0.293546)
64:	1104.69	0.322877	(-0.507693	0.254267	-0.021763)
65:	1123.92	8.063681	(2.320282	1.626605	-0.184737)
66:	1150.71	13.947298	(3.591570	-0.008558	-1.023646)
67:	1163.51	34.045253	(5.220003	1.100615	-2.363360)
68:	1170.31	0.207020	(0.128456	-0.356001	0.252552)
69:	1223.86	3.484796	(1.775545	0.549683	0.173450)
70:	1237.79	125.468801	(10.117778	4.801591	-0.209989)
71:	1251.70	14.630482	(-3.451610	-1.608975	0.357865)
72:	1260.75	24.035923	(-4.749064	-1.199007	0.211425)
73:	1275.50	1.236957	(0.944825	0.583839	-0.058273)
74:	1281.42	36.691711	(5.771841	1.820519	-0.251542)
75:	1294.34	0.127885	(0.306524	0.180172	-0.038289)
76:	1304.31	1.453032	(-0.646102	1.014045	-0.085418)
77:	1316.41	14.434786	(1.989177	3.197191	0.505897)
78:	1324.30	98.818925	(-8.676757	-4.850524	0.072273)
79:	1326.55	26.883666	(3.497393	3.827741	-0.017537)
80:	1333.70	1.065254	(0.625662	0.378203	-0.728536)
81:	1344.72	0.336414	(0.378952	0.201704	0.390031)
82:	1346.04	0.862417	(0.095853	0.923599	-0.013916)
83:	1354.21	0.610497	(-0.122424	-0.732228	-0.243622)
84:	1357.14	0.282124	(-0.357195	-0.303159	0.250261)
85:	1359.79	0.479556	(0.440983	0.255412	-0.468886)
86:	1380.12	3.789679	(-1.796768	-0.744746	0.081575)
87:	1382.37	4.791722	(1.376565	1.701973	-0.008934)
88:	1403.99	7.653571	(2.471105	1.136292	-0.506018)
89:	1410.73	2.057119	(-1.425778	-0.155756	-0.003905)
90:	1414.84	0.115730	(-0.058998	-0.189780	-0.276103)
91:	1419.45	0.138651	(0.008904	0.067598	0.366063)
92:	1423.75	10.609956	(3.220934	-0.081331	-0.478463)
93:	1436.49	2.187201	(-1.271008	-0.338441	-0.676165)
94:	1481.64	2.393461	(0.535143	-1.320686	0.602388)
95:	1489.82	0.769971	(-0.680996	0.195299	-0.517758)
96:	1490.83	2.951495	(-0.097380	-1.630835	-0.531403)
97:	1490.97	0.108718	(-0.297887	-0.026367	-0.138876)
98:	1493.14	1.115953	(0.730942	-0.112239	0.754374)
99:	1495.10	0.670594	(-0.793339	0.187779	-0.077114)
100:	1498.84	0.321514	(-0.534756	0.161002	-0.098124)
101:	1506.32	0.664670	(0.148136	-0.757712	-0.261914)
102:	1512.17	3.798044	(0.527733	-1.674707	0.845516)
103:	1513.47	6.258450	(-0.412882	-1.417823	2.019345)
104:	1515.18	1.392254	(1.071709	-0.288225	0.400774)
105:	1520.20	8.981652	(1.036967	2.165533	1.793549)
106:	1613.83	470.934238	(20.567886	-2.839100	-6.311561)
107:	1653.67	9.447273	(-3.070820	-0.062693	-0.115790)
108:	1727.56	136.145954	(-4.348315	10.775020	-1.066325)
109:	2842.89	4.155474	(1.927574	-0.304132	0.589436)
110:	2844.39	3.481349	(1.776691	-0.466242	0.327620)
111:	2845.18	4.326214	(1.948091	-0.357696	0.634988)
112:	2848.50	0.415404	(0.053482	0.477396	-0.429694)
113:	2849.73	0.832463	(-0.467842	0.745379	0.240826)
114:	2853.77	9.136376	(-0.326798	-2.955208	-0.544355)
115:	2856.96	10.699042	(-1.636053	2.832377	-0.003521)
116:	2859.57	26.901965	(-1.670867	2.016747	-4.476929)
117:	2860.42	56.621836	(-6.133148	3.161225	-3.002165)

118:	2863.55	157.758592	(6.660319	9.053342	5.606759)
119:	2868.88	13.564817	(2.275045	-1.951090	-2.140616)
120:	2868.96	2.457602	(0.221869	1.284704	0.870581)
121:	2874.36	6.876179	(1.029736	1.300129	2.031130)
122:	2876.95	8.722840	(0.209605	2.904470	0.492908)
123:	2879.56	8.875800	(-0.385261	0.427512	-2.923116)
124:	2886.17	4.386866	(0.882133	0.671136	1.777156)
125:	2890.18	6.744894	(0.053351	-0.745656	2.487176)
126:	2905.30	1.959359	(0.000692	0.817809	-1.136022)
127:	2916.85	99.113039	(-2.983967	2.487125	9.166416)
128:	2917.63	48.840036	(-1.552198	6.049545	3.135877)
129:	2921.23	36.878943	(-1.266413	-5.648491	1.835671)
130:	2926.00	155.489496	(-3.187936	-6.802208	9.952715)
131:	2931.77	10.599849	(1.170013	3.018655	-0.344439)
132:	2947.67	4.185297	(0.719830	-0.381652	-1.876562)
133:	3089.99	13.377090	(1.077050	-3.304957	1.137678)
134:	3135.59	1.237003	(-0.124121	1.075308	-0.255557)

The first frequency considered to be a vibration is 6
The total number of vibrations considered is 129

TMA

Mode	freq (cm**-1)	T**2	TX	TY	TZ
6:	85.30	1.718799	(-0.086239	0.434462	1.233939)
7:	120.93	2.886238	(-0.183780	1.613766	0.498219)
8:	142.04	1.773459	(0.965480	0.545289	-0.737541)
9:	194.61	1.205479	(-0.890502	-0.340110	0.544803)
10:	203.74	3.543077	(-0.075807	-0.207304	-1.869319)
11:	228.95	1.414320	(-0.318330	0.314753	-1.101779)
12:	275.81	2.767159	(0.547549	-1.319411	0.852352)
13:	348.15	2.292951	(0.758635	-1.164320	-0.601484)
14:	414.36	6.267083	(-0.415347	2.033985	-1.399099)
15:	445.27	2.966034	(-1.519197	-0.022617	-0.810902)
16:	452.29	11.937683	(-1.142638	-3.247387	0.294172)
17:	548.51	7.269649	(-1.561512	2.122822	0.570049)
18:	572.65	4.019440	(-0.621685	-1.864713	-0.394706)
19:	616.25	17.985227	(-3.166150	-2.773066	0.520408)
20:	658.74	1.657603	(-0.510322	1.180578	-0.058406)
21:	692.51	17.991664	(1.546096	3.750299	1.239558)
22:	694.40	8.202146	(1.599148	1.781516	-1.571965)
23:	755.78	47.290108	(-0.084663	1.639785	6.677877)
24:	764.02	9.973900	(-1.500077	1.216295	-2.498859)
25:	776.15	2.858419	(0.726594	-1.223363	-0.913161)
26:	802.61	18.038463	(-1.412230	-3.956638	-0.623768)
27:	825.71	5.011175	(0.221010	2.214204	-0.244194)
28:	875.67	8.460037	(-1.246441	1.930476	-1.783167)
29:	905.93	64.371007	(5.244688	1.837368	-5.786910)
30:	924.64	66.694363	(2.807096	-3.192301	-6.973076)
31:	947.76	19.449747	(1.027608	-0.478648	-4.262003)
32:	1004.61	2.666234	(-0.993445	-1.295218	-0.041371)
33:	1043.91	116.314190	(-9.865320	-4.348103	0.289217)
34:	1066.11	63.571565	(6.199967	-4.972395	0.638167)
35:	1121.33	56.115720	(-6.496614	-3.695679	0.501684)
36:	1161.01	24.364899	(4.839376	0.934610	-0.268033)
37:	1204.21	164.593512	(10.795956	6.847681	-1.072433)
38:	1249.23	391.359672	(-12.215940	-15.426562	2.037569)
39:	1290.05	117.715105	(-4.235570	-9.902056	1.313137)
40:	1317.35	58.730533	(-3.604192	6.742427	-0.529166)
41:	1356.44	47.065625	(2.990666	6.170914	0.203377)
42:	1373.84	151.477150	(3.570468	-11.771252	0.408085)
43:	1429.53	40.208586	(-0.847614	-6.062556	1.653951)
44:	1509.64	15.551878	(-0.372408	-3.904547	0.409519)
45:	1591.90	197.835821	(8.969875	-4.936931	9.643851)
46:	1615.82	60.919624	(-5.200102	4.679044	-3.461952)
47:	1653.05	7.452678	(2.207171	1.243352	1.017423)
48:	1680.61	578.648972	(24.037844	0.902932	-0.125421)
49:	1759.86	194.452669	(0.062765	-13.878171	1.358343)
50:	3005.06	151.042770	(-5.008413	-10.769667	3.157980)
51:	3186.49	0.308837	(0.249021	-0.438486	0.233570)
52:	3190.14	1.226682	(0.964871	-0.521341	0.154623)
53:	3198.41	0.204461	(-0.000432	-0.417585	-0.173446)

The first frequency considered to be a vibration is 6
The total number of vibrations considered is 48

TMA and NPG

Mode	freq (cm**-1)	T**2	TX	TY	TZ
6:	34.59	0.407101	(-0.301513	0.553004	-0.101871)
7:	48.66	0.342561	(0.480069	-0.192763	0.273747)
8:	49.24	0.320507	(-0.331229	-0.369142	0.273000)
9:	68.01	0.699190	(0.481768	0.189851	-0.656541)
10:	74.33	0.347965	(0.524316	0.017752	-0.269710)
11:	88.16	0.315510	(-0.468430	-0.117017	-0.287037)
12:	89.41	0.096129	(0.242144	0.180811	0.069298)
13:	99.75	1.595050	(-1.210327	-0.244050	-0.265703)
14:	109.77	0.195020	(0.175772	-0.137843	0.380951)
15:	119.74	0.559538	(-0.525222	-0.466977	-0.256149)
16:	129.62	4.125671	(0.836173	1.449581	-1.151173)
17:	146.40	1.362147	(-0.665459	0.914832	0.287041)
18:	154.93	3.008243	(-0.280178	-1.710059	-0.073760)
19:	158.48	1.068490	(-0.893890	0.467012	-0.226604)
20:	181.48	2.866517	(1.407368	0.137202	0.931133)
21:	187.02	6.221860	(1.697063	0.947501	1.563355)
22:	207.29	0.474789	(0.204343	-0.048464	-0.656265)
23:	217.77	4.119257	(-1.124066	-1.645828	0.383381)
24:	231.52	0.635721	(0.785927	-0.099354	-0.090376)
25:	255.59	0.910489	(-0.812428	0.495802	-0.068051)
26:	274.35	1.893607	(0.494147	0.386649	-1.224715)
27:	289.70	6.284296	(-0.414234	2.337400	0.805770)
28:	297.87	1.682287	(-1.181113	-0.534744	0.036173)
29:	330.68	2.423950	(1.485028	-0.456272	-0.102269)
30:	339.97	2.825032	(-0.426596	1.625253	-0.039999)
31:	354.17	2.145017	(1.243483	-0.755178	-0.168735)
32:	369.72	9.655429	(0.358555	2.578302	-1.696829)
33:	378.58	6.202738	(0.532360	-2.288527	0.825818)
34:	391.57	2.174252	(0.983194	-0.673265	0.868502)
35:	408.86	0.571471	(0.560065	0.497701	-0.100459)
36:	419.39	4.544456	(-0.694855	2.001672	-0.234400)
37:	430.84	11.245806	(-2.408198	-2.088260	1.041902)
38:	435.76	24.093452	(1.273359	4.569518	-1.261552)
39:	441.41	13.570756	(-1.149354	-2.808431	2.088649)
40:	444.52	0.388303	(-0.428857	-0.040442	0.450277)
41:	454.65	17.738007	(2.209593	3.034661	-1.909592)
42:	459.15	2.318619	(-0.473363	-1.023553	1.023174)
43:	464.20	6.635404	(-0.733092	2.460281	0.212130)
44:	464.87	0.976756	(-0.887349	-0.355829	-0.250506)
45:	470.79	84.392771	(-4.468463	-7.913627	1.341684)
46:	472.78	3.807416	(0.357426	-0.356327	1.884859)
47:	509.32	14.183697	(1.210288	2.209761	-2.799260)
48:	548.52	71.762379	(-5.086816	-6.555230	1.707526)
49:	592.13	113.115700	(-4.547442	-9.416245	1.941858)
50:	604.88	10.632892	(1.303273	2.833508	0.951632)
51:	616.28	14.777113	(-0.131214	-3.770918	0.734896)
52:	623.72	6.003181	(-2.391338	0.518169	-0.127220)
53:	636.31	4.784077	(1.084433	-0.816893	1.714867)
54:	678.25	86.693223	(4.525545	-8.125394	0.436628)
55:	682.36	96.489182	(1.095162	9.185170	-3.304914)
56:	689.26	37.291755	(-1.477845	-2.631309	5.308855)
57:	707.43	21.349985	(-0.376632	-4.544222	-0.747111)
58:	713.65	75.952226	(-4.512283	5.838625	4.637023)
59:	731.23	137.533551	(0.752964	8.872857	-7.631448)
60:	740.25	7.408597	(1.417204	-2.214861	0.703224)
61:	748.43	24.216197	(-3.820845	2.134064	2.250135)
62:	778.14	34.297176	(2.641243	4.405711	-2.812601)
63:	789.47	74.309942	(8.583641	0.271845	-0.746430)
64:	796.43	75.909645	(8.261391	-2.236006	-1.630748)
65:	816.03	19.658707	(1.896850	-4.005211	-0.137682)
66:	871.54	315.787854	(3.619815	17.392539	0.429401)
67:	879.04	11.440096	(1.527291	1.281112	-2.732440)
68:	897.06	1.022811	(0.910154	0.431937	0.088666)
69:	910.99	20.147613	(3.736748	-2.388252	0.693236)
70:	917.28	0.790765	(-0.374810	0.736290	-0.328877)
71:	920.85	1.461399	(-1.156436	0.298409	0.187102)
72:	927.75	31.570360	(3.671056	2.999626	-3.015950)
73:	973.63	102.949074	(-8.232609	5.928267	0.169932)
74:	983.30	9.882021	(-2.068721	-2.306542	-0.531302)
75:	994.88	2.472945	(-1.389332	0.668414	-0.309717)
76:	1007.10	12.130834	(1.844181	-2.890408	-0.612675)

77:	1015.38	0.593129	(-0.626600	0.431164	-0.120825)
78:	1018.39	0.327553	(-0.219184	0.103915	-0.518376)
79:	1028.03	4.222530	(-1.246098	1.632808	-0.060909)
80:	1030.06	1.801238	(-1.192200	0.465071	0.404483)
81:	1077.86	88.293456	(-8.366688	3.233145	-2.799778)
82:	1091.93	106.846924	(-4.532886	-9.289758	0.016411)
83:	1097.24	93.363212	(5.598945	7.805092	-1.046692)
84:	1100.79	102.863389	(-7.802500	6.393665	-1.051393)
85:	1108.54	165.194813	(-8.754976	9.369826	-0.866932)
86:	1114.76	96.583901	(-5.379548	-8.137751	-1.192213)
87:	1121.79	90.370339	(-4.997176	-7.900574	-1.726123)
88:	1149.38	13.434004	(1.063245	3.482016	0.423177)
89:	1151.14	88.037771	(0.676889	-9.358281	0.046492)
90:	1180.62	159.142867	(-11.057948	-6.034953	-0.666331)
91:	1192.03	143.850520	(-0.222102	-11.988993	0.255413)
92:	1207.42	4.181203	(1.598743	-1.274195	-0.040632)
93:	1218.11	89.745712	(-4.297455	8.253425	1.777235)
94:	1221.20	2.457735	(0.120624	-1.495359	0.455066)
95:	1236.29	0.818869	(0.838043	0.014278	0.341100)
96:	1242.11	16.164247	(1.294889	3.753250	-0.632951)
97:	1251.38	3.955814	(-1.473133	-1.325356	-0.170662)
98:	1254.04	3.904482	(1.359939	-1.400100	-0.307843)
99:	1278.95	9.918434	(2.919681	-0.287931	-1.144987)
100:	1284.06	2.941889	(0.045765	0.416859	-1.663137)
101:	1330.39	0.930770	(-0.135161	-0.813882	-0.500098)
102:	1332.66	215.932436	(13.186296	6.136641	2.096583)
103:	1341.81	14.673876	(-3.492843	0.821466	-1.341312)
104:	1355.76	6.219677	(-1.306780	1.972964	-0.787031)
105:	1392.02	3.625600	(-0.037793	1.040903	1.593955)
106:	1411.27	4.180797	(1.788199	0.554603	0.821922)
107:	1425.33	14.281309	(-3.334345	-0.260094	-1.759490)
108:	1425.63	7.185617	(1.645448	1.854834	1.018680)
109:	1431.33	7.572195	(-1.333676	1.205524	2.083318)
110:	1432.61	11.614982	(-1.079488	-0.335260	3.215166)
111:	1439.20	13.297750	(2.023551	-1.906172	2.359978)
112:	1460.76	6.452170	(0.286562	-2.475481	-0.491982)
113:	1462.90	7.949509	(1.914052	-1.635624	1.269113)
114:	1471.46	40.220624	(-3.387498	-5.317282	-0.687016)
115:	1471.68	49.865750	(6.858559	-1.483249	-0.791132)
116:	1497.69	1.949393	(0.672010	0.620316	1.054990)
117:	1502.24	0.956162	(-0.635780	0.396036	0.628571)
118:	1508.35	12.114763	(-2.496514	-2.355856	0.576300)
119:	1517.12	3.454573	(0.367252	1.638722	0.796422)
120:	1517.53	5.110424	(-2.014310	0.343888	-0.966809)
121:	1517.98	9.823501	(0.706444	2.876478	1.024848)
122:	1519.92	9.933767	(-2.232633	2.187831	-0.403127)
123:	1523.63	1.166904	(0.549572	-0.750817	0.548770)
124:	1524.42	1.227591	(0.692994	-0.839419	-0.206706)
125:	1527.43	0.903429	(-0.697038	-0.640484	0.085719)
126:	1537.28	24.269002	(-4.919131	-0.202855	0.173219)
127:	1540.91	9.356389	(2.410741	1.261342	-1.397759)
128:	1542.88	26.098884	(2.340552	4.347266	1.312241)
129:	1594.47	108.346878	(-3.486734	-2.493034	-9.485481)
130:	1600.65	61.974354	(4.178511	-0.176185	6.669585)
131:	1649.04	157.086788	(2.561380	-9.978396	7.138469)
132:	1652.13	275.017304	(7.598708	-11.961647	-8.613707)
133:	2816.86	63.845270	(7.606118	-1.028392	-2.221407)
134:	2822.22	48.328446	(-4.674033	-1.284854	4.983073)
135:	2862.65	24.272028	(0.870494	-0.919493	-4.761176)
136:	2862.82	46.336167	(1.628461	2.169965	6.243039)
137:	2867.27	36.405889	(-5.384718	-2.505342	1.064877)
138:	2868.40	2.442257	(-1.323255	0.486415	-0.674280)
139:	2871.21	8.198309	(-0.430823	-1.400188	2.460117)
140:	2874.25	18.803518	(0.149700	0.497397	4.305079)
141:	2877.29	54.931016	(5.126712	3.276245	4.232501)
142:	2881.19	23.585827	(2.789952	-3.158107	2.414198)
143:	2901.48	22.275540	(2.959022	1.731869	3.243511)
144:	2915.27	22.115990	(4.372191	1.076680	-1.356721)
145:	2917.21	20.264849	(-1.880238	0.317274	4.077854)
146:	2918.89	5.293766	(-1.785363	0.795284	-1.213989)
147:	2921.03	40.605886	(5.862063	2.240344	1.105875)
148:	2921.58	31.763571	(-3.454799	-4.449366	0.176287)
149:	2921.86	6.647004	(-1.953827	-1.568648	-0.607377)
150:	2923.99	35.239563	(-4.415760	3.954814	-0.316352)
151:	2924.47	42.475943	(1.794451	-2.853647	5.577866)
152:	2927.01	21.444636	(0.859505	-4.475398	-0.822621)
153:	3173.81	0.191268	(0.161205	-0.373417	-0.160749)
154:	3180.94	0.324333	(-0.338764	0.407090	-0.209402)
155:	3188.38	0.187303	(-0.167226	-0.027099	0.398252)

156:	3421.91	38.448170	(	4.015726	-1.646132	4.428585)
157:	3423.51	38.119433	(	1.625163	4.027180	4.388633)
158:	3437.33	165.712469	(	8.764851	8.344253	4.388996)

The first frequency considered to be a vibration is 6
The total number of vibrations considered is 153

TMA and HMMM

Mode	freq (cm**-1)	T**2		TX	TY	TZ
6:	13.13	0.161189	(	0.136915	0.260289	-0.273300)
7:	14.90	1.282254	(	-1.058693	0.307329	-0.258790)
8:	19.83	0.122227	(	-0.210267	0.248835	0.126871)
9:	27.98	0.934362	(	0.632206	0.610969	0.401739)
10:	33.20	1.405469	(	-0.844508	0.027991	-0.831560)
11:	33.43	0.495519	(	0.123061	0.460983	0.517562)
12:	37.38	1.313776	(	0.690720	0.603142	0.687679)
13:	48.62	2.059650	(	0.093983	0.469711	-1.352845)
14:	56.38	1.334951	(	-0.401727	-0.910981	-0.586242)
15:	60.49	2.603556	(	-0.314839	-1.578982	-0.106060)
16:	78.26	2.129143	(	-0.176763	1.271961	0.692830)
17:	84.66	2.885257	(	0.467542	-1.590042	0.372061)
18:	92.21	0.012312	(	-0.051670	-0.062862	0.075434)
19:	98.42	4.771276	(	-1.873118	-0.917319	0.649023)
20:	121.79	0.387536	(	0.409078	-0.282392	-0.374762)
21:	132.85	1.872946	(	-0.183690	1.353139	0.090652)
22:	138.36	1.433921	(	0.742770	-0.880190	-0.327839)
23:	141.66	0.631938	(	0.675641	-0.410103	-0.085223)
24:	151.59	0.754526	(	0.450536	0.103694	0.735385)
25:	160.32	0.062456	(	-0.180849	-0.149565	-0.085906)
26:	162.30	2.788775	(	1.479169	-0.773824	-0.045061)
27:	170.79	1.406653	(	-0.657351	-0.791806	-0.589565)
28:	175.87	1.167449	(	-0.275145	0.903919	-0.524093)
29:	190.67	0.293090	(	0.381167	-0.063477	0.379174)
30:	198.59	0.595801	(	-0.386328	0.634817	0.208708)
31:	201.21	1.159865	(	-0.334441	0.060492	1.021937)
32:	229.66	5.093888	(	-0.764041	2.060697	0.513478)
33:	236.83	1.050740	(	-0.155412	0.986233	0.232233)
34:	248.85	3.941438	(	1.253142	1.091260	-1.086381)
35:	277.56	10.477151	(	-1.959607	-2.570362	0.174157)
36:	293.08	9.167832	(	1.045839	2.577480	-1.196098)
37:	302.27	2.574749	(	-1.455192	-0.562270	0.375523)
38:	310.50	1.746166	(	1.136825	-0.666688	0.096558)
39:	328.39	4.454687	(	-1.605632	-0.815176	-1.100964)
40:	349.86	1.352573	(	-0.612156	-0.070231	0.986360)
41:	394.53	15.353402	(	-1.834755	2.366303	-2.527387)
42:	404.75	5.540373	(	-1.232553	-1.989577	0.250540)
43:	410.26	8.558053	(	-2.660202	0.022480	1.216912)
44:	413.32	3.751257	(	-0.248473	-1.766626	-0.754024)
45:	434.97	2.927934	(	0.328981	-1.678023	0.062815)
46:	447.21	8.622485	(	1.626017	2.423377	0.325262)
47:	484.36	26.275542	(	1.447916	-4.909426	-0.276806)
48:	491.41	45.707417	(	-6.638600	-1.091016	0.667905)
49:	513.44	74.673654	(	1.543027	8.439707	1.031536)
50:	518.84	21.411365	(	3.887177	-2.436899	-0.602281)
51:	529.78	17.960097	(	3.051090	-0.722900	-2.851028)
52:	581.38	43.770393	(	1.427339	-5.445972	-3.474837)
53:	588.90	39.348531	(	6.232394	0.488254	-0.517109)
54:	612.27	62.622471	(	-6.057937	-4.927910	1.280458)
55:	626.10	13.463654	(	-1.874507	2.996627	0.984937)
56:	639.42	29.850309	(	-3.740808	1.743486	-3.580073)
57:	640.66	6.066001	(	-2.170582	-0.986358	-0.617795)
58:	656.27	21.132167	(	4.088366	1.941906	0.804012)
59:	688.39	16.467824	(	3.420594	1.574011	1.513224)
60:	721.72	47.638831	(	-2.589018	-6.348101	-0.798392)
61:	746.82	23.427125	(	4.421981	-1.091188	-1.637840)
62:	758.59	76.397638	(	-2.964012	8.145246	1.125717)
63:	759.57	38.907338	(	-0.506979	-6.078662	1.303907)
64:	775.10	13.751521	(	1.067876	-3.550844	-0.051680)
65:	795.16	41.013536	(	-1.185616	-5.693618	-2.681523)
66:	804.82	42.694821	(	-5.381669	-2.447015	-2.782907)
67:	821.33	146.571219	(	5.652427	-10.571349	-1.693479)
68:	823.80	35.640657	(	-0.237605	5.915678	-0.767431)
69:	843.25	75.033372	(	6.179935	4.506822	-4.065751)
70:	870.43	138.932096	(	9.511366	5.542697	4.212424)
71:	926.34	4.246987	(	0.391808	-1.935568	0.589110)
72:	950.10	148.592954	(	0.192983	-12.183762	-0.334139)

73:	961.99	35.679723	(5.650722	1.912958	-0.299435)
74:	974.41	6.862378	(-1.580108	2.005831	-0.585047)
75:	989.33	7.654877	(-1.098305	-2.406559	0.810602)
76:	998.53	3.552529	(-0.988676	-1.574629	-0.309181)
77:	1002.94	4.048553	(-0.580933	-1.923107	0.112822)
78:	1021.75	33.856714	(-4.635574	-1.289498	-3.271905)
79:	1023.78	13.696337	(-2.505449	-2.609556	-0.780564)
80:	1025.72	89.209655	(-9.174447	-1.493238	-1.676132)
81:	1030.53	112.796750	(6.202020	-7.988989	-3.241565)
82:	1056.42	84.491399	(6.023390	-6.562118	-2.269092)
83:	1059.90	52.478422	(1.720222	5.320706	-4.605360)
84:	1064.98	156.203896	(9.589724	7.511278	-2.796748)
85:	1089.99	15.007106	(1.507672	2.464346	-2.580897)
86:	1118.31	176.950431	(0.938491	9.257757	-9.505977)
87:	1134.50	45.475978	(4.931845	2.668510	3.745923)
88:	1155.72	2.379525	(-1.340476	-0.725560	-0.237087)
89:	1164.03	1.749844	(-1.239899	0.333431	-0.318304)
90:	1168.64	0.747411	(-0.545546	0.570116	-0.353211)
91:	1169.32	3.004979	(-1.506548	-0.828525	-0.220997)
92:	1170.20	2.104734	(-0.721093	-0.651951	-1.076902)
93:	1172.20	0.606512	(0.314372	0.231569	0.673838)
94:	1174.29	10.145439	(-2.023442	2.454714	-0.159696)
95:	1194.21	1.522467	(-0.289838	0.285664	-1.164842)
96:	1204.02	78.329515	(-3.559677	3.067281	-7.500000)
97:	1211.58	3.807740	(1.279834	-0.651315	1.321193)
98:	1213.32	7.187596	(0.814074	-2.491985	0.561150)
99:	1216.16	7.093087	(-1.399579	-2.235559	0.369518)
100:	1217.19	2.427853	(-0.488226	0.847872	1.212684)
101:	1217.55	3.616258	(-0.201009	0.655070	1.773905)
102:	1253.99	326.450512	(7.799510	13.951507	-8.424583)
103:	1279.08	8.412372	(-0.182695	-2.827831	0.618357)
104:	1303.12	10.461004	(-2.304699	-2.204779	-0.536949)
105:	1329.22	29.427496	(-4.235830	-3.051778	1.473735)
106:	1361.65	77.491516	(1.971699	-4.032805	7.572345)
107:	1382.11	464.463808	(-18.859678	-9.599868	-4.076625)
108:	1392.41	499.302628	(10.186529	-19.739837	-2.424061)
109:	1454.52	13.579023	(-2.611272	-2.598692	-0.084166)
110:	1458.09	3.833056	(-0.202021	-1.916499	-0.345361)
111:	1459.90	13.670340	(3.403132	1.437895	0.146603)
112:	1461.28	1.483685	(-0.370445	0.949265	-0.667346)
113:	1462.13	2.736564	(-0.736242	-0.916984	-1.163465)
114:	1464.11	3.574480	(-1.166899	-1.431508	-0.404489)
115:	1476.33	10.272446	(-2.625874	1.835190	-0.096486)
116:	1477.55	3.762603	(-0.249767	-0.327991	-1.895426)
117:	1478.53	7.222128	(-0.195276	-1.035860	2.472042)
118:	1481.28	7.691186	(-1.554904	-2.292343	0.136462)
119:	1481.63	8.357907	(2.396277	0.414512	1.563312)
120:	1484.61	5.046733	(-0.984625	-1.491538	-1.361088)
121:	1499.65	5.714187	(-1.623478	0.785125	-1.569103)
122:	1500.48	10.525525	(-1.839006	2.378909	1.218348)
123:	1502.77	9.515399	(2.352892	-0.329256	1.967457)
124:	1507.66	3.185886	(-1.536020	0.071887	-0.906290)
125:	1508.45	2.544262	(0.983460	0.962818	-0.806256)
126:	1520.13	0.561147	(0.381795	0.634261	-0.114417)
127:	1576.92	649.640076	(-24.457194	-6.231714	-3.556895)
128:	1595.56	704.485436	(3.501033	-26.159293	-2.814178)
129:	1608.16	3.061228	(0.280817	-0.421134	-1.674818)
130:	1636.59	18.206602	(3.888559	1.186407	1.295434)
131:	1788.31	401.123223	(-16.835441	-10.727293	1.617507)
132:	1823.97	305.803854	(1.120095	12.995374	11.647725)
133:	3013.83	26.988651	(-2.438347	4.557614	-0.520839)
134:	3015.12	27.106107	(-5.183022	0.403423	0.282199)
135:	3018.84	76.342284	(-4.034533	-7.260134	2.712062)
136:	3020.76	67.698555	(5.178062	-3.558859	-5.312321)
137:	3033.67	32.617331	(-4.750514	2.324821	2.155262)
138:	3081.47	26.892238	(-0.950300	4.334249	-2.683924)
139:	3081.74	27.753219	(3.914197	2.870468	2.047607)
140:	3086.37	26.476647	(1.289771	-4.842398	-1.168039)
141:	3091.07	24.850873	(4.908651	-0.819794	0.289750)
142:	3110.95	14.132421	(2.643237	1.866270	1.913832)
143:	3117.47	13.860611	(-2.128868	-0.974349	-2.894681)
144:	3118.72	11.210113	(-0.919119	-2.939973	1.312210)
145:	3121.07	15.325136	(1.888336	0.091372	3.427969)
146:	3122.01	15.492564	(-2.458262	1.441658	2.714983)
147:	3124.95	12.614890	(0.693321	-2.609697	2.307309)
148:	3169.39	4.543779	(1.888031	0.757717	-0.636382)
149:	3182.70	5.081352	(1.342388	0.108804	1.807625)
150:	3191.78	6.682592	(0.915337	1.525440	-1.875576)
151:	3200.08	5.665525	(-0.308461	-0.756560	2.235619)

```
152:      3752.21  107.756670  ( -7.172348   0.044425  -7.504141)

      The first frequency considered to be a vibration is 6
        The total number of vibrations considered is 147
```
