# Supplementary material for: Computationally-accelerated prediction of polyester-melamine coatings degradation to design sustainable organically-coated steels for outdoor applications
Source: RSC Adv. 2024 Jun 24;14(26):18343–54. doi: 10.1039/d3ra06744k (PMC11194856; doi:10.1039/d3ra06744k)
Supplement: RA-014-D3RA06744K-s003 [file RA-014-D3RA06744K-s003.pdf]

Electronic Supplementary Material (ESI) for RSC Advances.
This journal is © The Royal Society of Chemistry 2024

This ESI covers the method used to build the molecules and identify their ground states.

Molecule design and ground state identification took place over several stages and utilised Chemsketch, Avogadro, and ORCA calculations.

1. Each molecule was designed in 2D via chemsketch software. The software has an in-built ability to then visualise the structures in 3 dimensions. This was then saved as a .mol file.
2. The .mol file was then opened using Avogadro, which has an auto-optimisation tool. This manipulates the molecule in 3-dimensional space to find the orientation with the lowest energy levels. The Unified Force Field (UFF) was utilised to facilitate the identification of the lowest energy orientation. This force field was chosen as it is a full periodic force field that is commonly used in molecular dynamics and simulations.
    a. The 3D structure is manipulated until the lowest energy state for the structure is identified.
3. Finally, within the ORCA calculation, self-consistent field (SCF) calculations were performed.
    a. Utilising the SOSCF (second-order SCF) to achieve a quadratic convergence in orbital optimisation.
    b. The lowest stable energy state of each model is identified through convergence of the SCF calculation, which occurs after a number of calculation iterations.
