# Supplementary material for: Computationally-accelerated prediction of polyester-melamine coatings degradation to design sustainable organically-coated steels for outdoor applications
Source: RSC Adv. 2024 Jun 24;14(26):18343–54. doi: 10.1039/d3ra06744k (PMC11194856; doi:10.1039/d3ra06744k)
Supplement: RA-014-D3RA06744K-s004 [file RA-014-D3RA06744K-s004.pdf]

Electronic Supplementary Material (ESI) for RSC Advances.
This journal is © The Royal Society of Chemistry 2024

Information regarding the accelerated weathering testing procedure.

The fluorescent UV bulbs utilised in this investigation were Type 1A (UVA-340) and simulate daylight between the ranges of 300nm to 340nm, they are the standard bulb of choice for the simulation of external light. Irradiance was set at 0.89W/m^2 for accelerated weathering tests and was maintained by the Q-lab Solar eye and periodic calibration. The samples were also moved to different locations within the sample holder racking following each period of analysis (250 hours) this was performed to ensure adequate UV exposure in the event of a lower irradiance spot forming within one of the bulbs during testing.

Care was taken during sample analysis to ensure that the sample coatings were not damaged, contaminated, or otherwise influenced. The samples were also returned to the machine in the same orientation that they were removed in.
