# Supplementary material for: Computationally-accelerated prediction of polyester-melamine coatings degradation to design sustainable organically-coated steels for outdoor applications
Source: RSC Adv. 2024 Jun 24;14(26):18343–54. doi: 10.1039/d3ra06744k (PMC11194856; doi:10.1039/d3ra06744k)
Supplement: RA-014-D3RA06744K-s005 [file RA-014-D3RA06744K-s005.pdf]

Electronic Supplementary Material (ESI) for RSC Advances.
This journal is © The Royal Society of Chemistry 2024

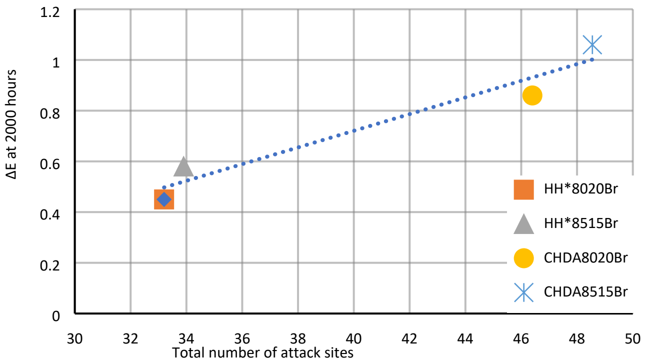

Figure 1 - Linear correlation between the number of molecular sites that would undergo nucleophilic or electrophilic attacks according to the dual descriptor of the Fukui function against the ΔE of samples exposed in accelerated weathering tests.

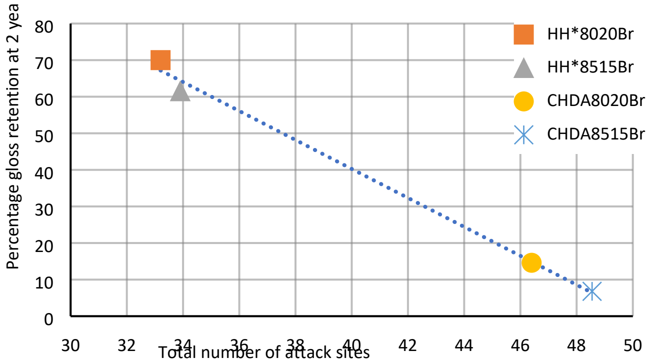

Figure 2 - Linear correlation between the number of molecular sites that would undergo nucleophilic or electrophilic attacks according to the dual descriptor of the Fukui function against the average percentage of gloss retention of samples exposed in natural weathering.

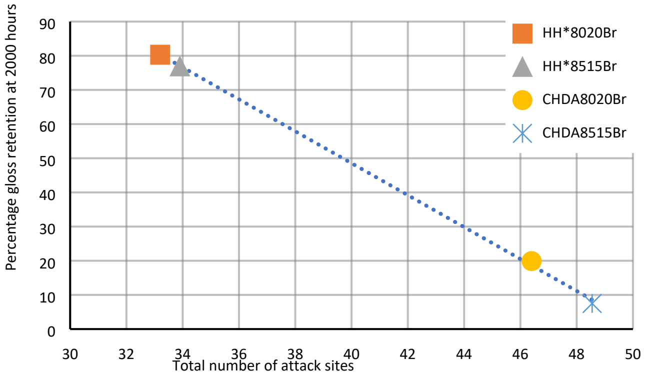

Figure 3 - Linear correlation between the number of molecular sites that would undergo nucleophilic or electrophilic attacks according to the dual descriptor of the Fukui function against the percentage of gloss retention of samples exposed in accelerated weathering tests.

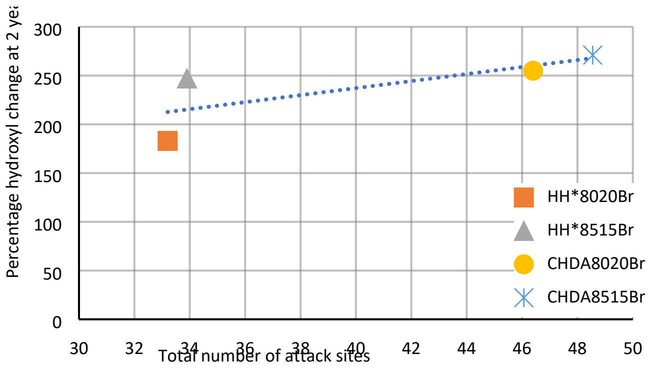

Figure 4 - Linear correlation between the number of molecular sites that would undergo nucleophilic or electrophilic attacks according to the dual descriptor of the Fukui function against the percentage change in hydroxyl activity of samples exposed in natural weathering tests.

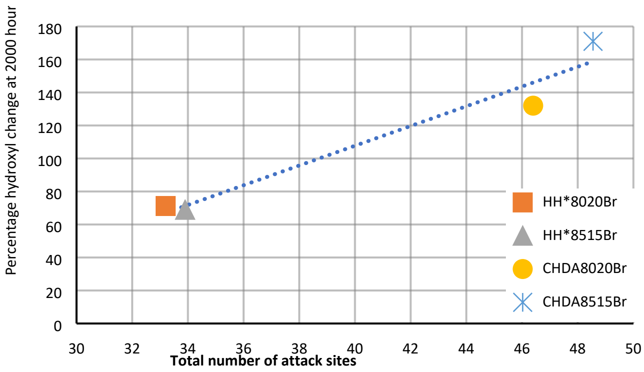

Figure 5 - Linear correlation between the number of molecular sites that would undergo nucleophilic or electrophilic attacks according to the dual descriptor of the Fukui function against the percentage change in hydroxyl activity of samples exposed in accelerated weathering tests.

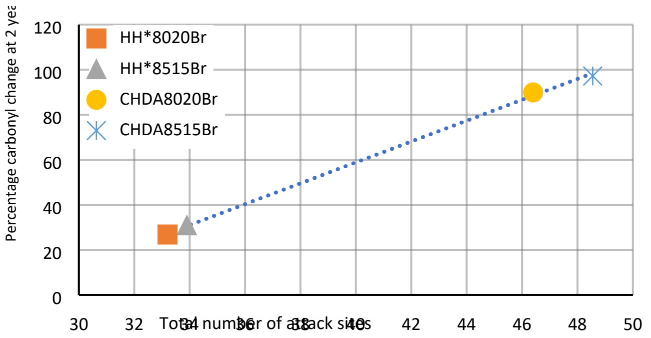

Figure 6 - Linear correlation between the number of molecular sites that would undergo nucleophilic or electrophilic attacks according to the dual descriptor of the Fukui function against the percentage change in carbonyl activity of samples exposed in natural weathering tests.

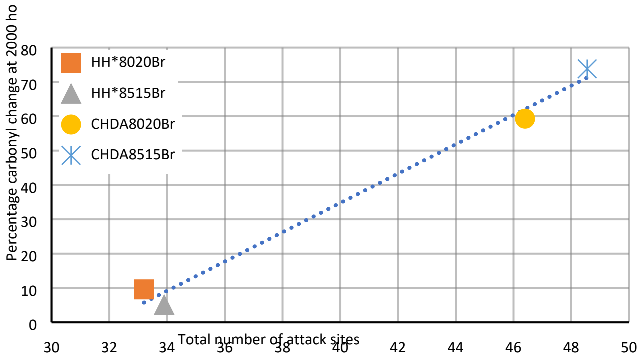

Figure 7 - Linear correlation between the number of molecular sites that would undergo nucleophilic or electrophilic attacks according to the dual descriptor of the Fukui function against the percentage change in carbonyl activity of samples exposed in accelerated weathering tests.
