# Supplementary material for: Computationally-accelerated prediction of polyester-melamine coatings degradation to design sustainable organically-coated steels for outdoor applications
Source: RSC Adv. 2024 Jun 24;14(26):18343–54. doi: 10.1039/d3ra06744k (PMC11194856; doi:10.1039/d3ra06744k)
Supplement: RA-014-D3RA06744K-s006 [file RA-014-D3RA06744K-s006.pdf]

Electronic Supplementary Material (ESI) for RSC Advances.
This journal is © The Royal Society of Chemistry 2024

To assess the influence of imaginary modes on the models they were compared against the newly generated models to calculate the RMSD values (**Table 1**) and compare the Fukui functions (**Table 2**).

Table 1 shows that there is a considerable correlation between the models, as all of the calculated values are below 0.2 Å, while it is commonly accepted that values below 1.5 Å show significant correlation.

Table 2 shows that for the majority of models that the presence of imaginary modes did not influence the number of electrophilic or nucleophilic attack sites and that in the instance where a difference was observed it was very small and likely to be associated with the isovalue used to visually explore the models. The observed differences also did not alter the initial rankings produced within this body of work.

Table 1 – *Breakdown of the determined RMSD values*

Model name	RMSD Value (Å)
**CHDA**	0.004
**HMMM CHDA**	0.197
**HHPA**	0
**HMMM HHPA**	0.002
**TMA**	0.002
**TMA NPG**	0.607
**HMMM TMA**	1.099
**DDSA**	0.708

Table 2 – *Comparing the number of attack sites between models with imaginary modes and without*

	**Before removal of imaginary modes**		**After removal of imaginary modes**		
**Model designation**	**Electrophilic attack site**	**Nucleophilic attack site**	**Electrophilic attack site**	**Nucleophilic attack site**	**Difference in number of sites**
**CHDA**	8	12	8	12	0
**HMMM CHDA**	6	6	6	6	0
**HHPA**	8	8	8	8	0
**HMMMM HHPA**	12	10	9	10	-3 electrophilic
**TMA**	10	18	10	18	0
**TMA NPG**	8	8	8	8	0
**HMMMM TMA**	16	16	16	16	0
**DDSA**	3	8	5	8	+2 electrophilic

Table 3 – *Weighted attack sites as a function of the polyester-melamine ratio*

Monomer base	Number of electrophilic sites	Number of nucleophilic sites	Total
**HHPA**	17.8	14.8	32.6
**CHDA**	22.8	23.6	46.4
**TMA**	24.8	35.2	60
**DDSA**	13.2	19.6	32.8
